# High conservation combined with high plasticity: genomics and evolution of *Borrelia bavariensis*

**DOI:** 10.1186/s12864-020-07054-3

**Published:** 2020-10-08

**Authors:** Noémie S. Becker, Robert E. Rollins, Kateryna Nosenko, Alexander Paulus, Samantha Martin, Stefan Krebs, Ai Takano, Kozue Sato, Sergey Y. Kovalev, Hiroki Kawabata, Volker Fingerle, Gabriele Margos

**Affiliations:** 1 Division of Evolutionary Biology, Faculty of Biology, LMU Munich, Grosshaderner Strasse 2, 82152 Planegg-Martinsried, Germany; 2 University of Helsinki, Biomedicum Helsinki, PO Box 63, Haartmaninkatu 8, FIN-00014 Helsinki, Finland; 3 Gene Center, Laboratory for Functional Genome Analysis, LMU Munich, Feodor-Lynen-Strasse 25, 81377 Munich, Germany; 4 Department of Veterinary Epidemiology, Joint Faculty of Veterinary Medicine, Yamaguchi University, Yamaguchi, 753–8515 Japan; 5 Department of Bacteriology-I, National Institute of Infectious Diseases, Tokyo, 162–8640 Japan; 6 Laboratory of Molecular Genetics, Institute of Natural Sciences and Mathematics, Ural Federal University, Lenin Avenue 51, Yekaterinburg, 620000 Russia; 7 National Reference Centre for Borrelia at the Bavarian Health and Food Safety Authority, Veterinärstr 2, 85764 Oberschleissheim, Germany

**Keywords:** *Borrelia bavariensis*, Lyme Borreliosis, Genome assembly, Plasmids, Genetic plasticity

## Abstract

**Background:**

*Borrelia bavariensis* is one of the agents of Lyme Borreliosis (or Lyme disease) in Eurasia. The genome of the *Borrelia burgdorferi* sensu lato species complex, that includes *B. bavariensis*, is known to be very complex and fragmented making the assembly of whole genomes with next-generation sequencing data a challenge.

**Results:**

We present a genome reconstruction for 33 *B. bavariensis* isolates from Eurasia based on long-read (Pacific Bioscience, for three isolates) and short-read (Illumina) data. We show that the combination of both sequencing techniques allows proper genome reconstruction of all plasmids in most cases but use of a very close reference is necessary when only short-read sequencing data is available. *B. bavariensis* genomes combine a high degree of genetic conservation with high plasticity: all isolates share the main chromosome and five plasmids, but the repertoire of other plasmids is highly variable. In addition to plasmid losses and gains through horizontal transfer, we also observe several fusions between plasmids. Although European isolates of *B. bavariensis* have little diversity in genome content, there is some geographic structure to this variation. In contrast, each Asian isolate has a unique plasmid repertoire and we observe no geographically based differences between Japanese and Russian isolates. Comparing the genomes of Asian and European populations of *B. bavariensis* suggests that some genes which are markedly different between the two populations may be good candidates for adaptation to the tick vector, (*Ixodes ricinus* in Europe and *I. persulcatus* in Asia).

**Conclusions:**

We present the characterization of genomes of a large sample of *B. bavariensis* isolates and show that their plasmid content is highly variable. This study opens the way for genomic studies seeking to understand host and vector adaptation as well as human pathogenicity in Eurasian Lyme Borreliosis agents.

## Background

The *Borrelia burgdorferi* sensu lato (s.l.) species complex contains over 20 genospecies of spirochetal bacteria, among them the agents of human Lyme Borreliosis (LB or Lyme disease). These bacteria are obligate parasites that are transmitted between hosts (mainly rodents and birds) by ticks of the genus *Ixodes* [1–5]⁠.

*Borrelia bavariensis* was raised to species level in 2009 and was thereby separated from its sister species *B. garinii* [6, 7]⁠. Both species are present across Eurasia; their main vectors are *Ixodes persulcatus* in Asia and *I. ricinus* in Europe and both are pathogenic to humans. However, the main hosts of the two species differ, with *B. bavariensis* being found in rodents, while its sister species *B. garinii* is found only in birds [7–9]⁠⁠. Originally, the two species were differentiated genetically by their so-called OspA type (i.e. allele at the gene sequence of the Outer Surface Protein A) [10]⁠ but more recent studies have confirmed their species status using multilocus sequence analyses (MLSA) for species delineation and phylogenies based on several genetic sequences [6, 11–13]⁠. *B. bavariensis* is of great interest as it has been isolated from many LB patients in Europe but isolates from questing ticks come almost exclusively from Asia ([6]⁠ and Margos, Fingerle, personal communication).

The members of *B. burgdorferi* s.l. are characterized by a very complex and fragmented genome that contains a main linear chromosome of approximately 900 kb and up to 20 different linear or circular plasmids whose repertoire vary between and within species [14–17]⁠. Plasmid types are defined based on the plasmid partition genes they contain, and in particular on the PFam32 gene sequence if present (described below). Each plasmid type can in turn be subdivided into sub-types based on organizational changes [14, 18]⁠. Several plasmids form families of related replicons (cp32 and lp28 families) that share long stretches of their sequences. This makes the reconstruction of *B. burgdorferi* s.l. genomes from Next-Generation Sequencing (NGS) data a challenge [18]⁠ and explains why, to date, only 34 fully assembled genomes can be found in NCBI [19]⁠ among which more than half (18) belong to the species *B. burgdorferi* sensu stricto (s.s.) that is the main LB pathogen in North America. A fully assembled genome is available for the species *B. bavariensis* for reference strain PBi [20]⁠ (Accession number: CP058872) and three strains that are still referenced as *B. garinii* in GenBank (BgVir CP003151.1 [21]⁠, SZ CP007564.1 [22]⁠ and NMJW1 CP003866.1 [23])⁠, but which are known to belong to the species *B. bavariensis* [11]⁠. However, for the latter, only the main chromosome (strains SZ, NMJW1 and BgVir) and two plasmids (strain BgVir only) are assembled.

The process of reconstruction of *B. burgdorferi* s.l. genomes can be facilitated by the identification of plasmid partition genes on assembled contigs. Five such genes have been described in *B. burgdorferi* s.s. and each replicon is believed to contain no more than one copy of these genes unless it is a fusion of two plasmids [24]⁠. In particular, the sequences of the protein family PFam32 are used to name plasmids in the different species of the complex based on the homology to the sequences in *B. burgdorferi* s.s.. Not all plasmids possess a PFam32 [25, 26]⁠ but PFam50 and 57/62 appear also to be unique for each plasmid type and allow for plasmid identification in such cases [26]⁠.

Genes encoded on plasmids play an important role in pathogenicity and infection of hosts and vectors [27–29]⁠. Description of the whole plasmid repertoire of different isolates from the same species is thus an important step in searching for genetic factors involved in host and vector adaptation. The species *B. bavariensis* is characterized by differentiation into two populations, one in Asia and one in Europe that utilize different vectors. Previous work has shown that the European population showed very little genetic variability on the main chromosome and on two plasmids and seemed to follow a clonal frame [11]⁠. In contrast, the Asian isolates described so far, showed higher genetic diversity (reviewed in [9]⁠)⁠. The origin of the species is still unknown, but this diversity pattern could suggest an Asian origin. In the present study, we combined long read (Pacific Bioscience, hereafter PacBio) and short read (Illumina) data to reconstruct the whole genome sequence of 33 *B. bavariensis* isolates from Europe and Asia (Table 1). We show that the plasmid content varies even in the European population, and that the genome of this species is for one part highly conserved and for the other part highly variable. 
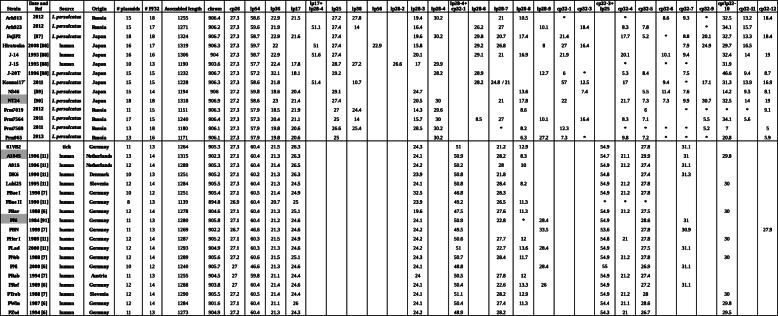

**Table 1:** Genome content of 33 *Borrelia bavariensis* strains

Strain	Date and Ref	Source	Origin	# plasmids	# PF32	Assembled length	chrom	cp26	lp54	lp36	lp17	lp17 + lp28-4	lp25	lp38	lp56	lp28-2	lp28-3	lp28-4	lp28-4 + cp32-1	lp28-6	lp28-7	lp28-8	lp28-9	cp32-1	cp32-3	cp32-3 + lp25	cp32-4	cp32-5	cp32-6	cp32-7	cp32-9	cp/lp32-10	cp32-11	cp32-12
**Arh913**	**2012**	***I. persulcatus***	**Russia**	15	18	1255	906.4	27.3	58.6	22.9	21.5		27.2	27.8			19.4	30.2			21	10.5		*			*		8.6	9.3	*	32.5	13.2	18.4
**Arh923**	**2012**	***I. persulcatus***	**Russia**	15	17	1271	906.2	27.3	59.6	21.9		51.1	27.4	14			16.4			26.2	27		10.1		18.4		8.3	7.8			*	34.1	15.7	
**FujiP2**	**[87]**	***I. persulcatus***	**Japan**	18	18	1324	906.7	27.3	58.7	22.9	21.6		27.4				19.6	30.2		29.8	20.7	17.4		21.4			17.7	5.2	*	8.8	20.1	32.7	13.3	18.4
**Hiratsuka**	**2008 [88]**	**human**	**Japan**	16	17	1319	906.3	27.3	59.7	22		51	27.4		22.9		15.8			29.2	26.8		8	27	16.4					7.9	24.9	29.7	16.5	
**J-14**	**1995 [88]**	**human**	**Japan**	16	16	1306	904	27.3	58.7	22.9		51.6	27.4				20.1			29.1	21	16.9		21.9			20.1		10.1	9.4		32.4	14	19
**J-15**	**1995 [88]**	**human**	**Japan**	10	13	1190	903.6	27.3	57.7	22.4	17.8		28.7	27.2		26.6	17	29.9									*		*	*		31.9		
**J-20T**	**1996 [88]**	***I. persulcatus***	**Japan**	15	15	1232	906.7	27.3	57.2	32.1	18.1		29.2					28.2		28.9			12.7	6	*		5.3	8.4		7.5		46.6	9.4	8.7
**Konnai17°**	**2011**	***I. persulcatus***	**Japan**	15	15	1228	906.3	27.3	58.6	21.8		51.4		10.7						28.2	24.8 / 21			57	12.5		17		9.4	*	17.1	31.3	13.9	16.9
**N346**	**[89]**	***I. persulcatus***	**Japan**	15	14	1194	906	27.2	59.8	18.6	20.4		29.1				24.7					13.6			7.4			5.5	11.4	7.6		14.2	9.3	8.1
**NT24**	**[90]**	***I. persulcatus***	**Japan**	18	18	1318	906.9	27.2	58.6	23	21.4		27.4				20.5	30			21	17.8		22			21.7	7.3	7.3	9.9	30.7	32.5	14	19
**Prm7019**	**2012**	***I. persulcatus***	**Russia**	11	15	1151	906.3	27.3	57.9	18.5	21.9		27	24.4			14.3	29.6				8.6						6		*	*	*	*	9.1
**Prm7564**	**2011**	***I. persulcatus***	**Russia**	17	15	1240	906.4	27.3	57.3	20.4	21.1		25	14			15.7	30		8.5	27		10.1		16.4		8.3	7.1			5.5	34.1	5.6	
**Prm7569**	**2011**	***I. persulcatus***	**Russia**	13	18	1180	906.1	27.3	57.9	19.8	20.6		26.6	25.4			28.5	30.2			*	8.2		12.3			*	*	*	*	5.2	7		5
**Prm965**	**2013**	***I. persulcatus***	**Russia**	13	16	1171	906.1	27.3	57.9	19.8	20.6		25					30.2				6.3	27.2	7.3	*		9.8	7.2	*	*	*	20.8		5.9
**61VB2**		**tick**	**Germany**	11	13	1264	905.3	27.3	60.4	21.5	26.3						24.3		51		21.2	12.9				54.9		27.8		31.1				
**A104S**	**1996 [11]**	**human**	**Netherlands**	13	14	1315	902.3	27.1	60.4	21.3	26.3						24.1		50.9		28.2	8.3				54.7	21.1	29.9		31		29.8		
**A91S**	**1996 [11]**	**human**	**Netherlands**	12	14	1289	905.3	27.3	60.4	21.4	26.5						24.2		50.2		28	10				54.9	21.2	27.4		31.1				
**DK6**	**1990 [11]**	**human**	**Denmark**	10	13	1251	905.2	27.1	60.2	21.3	26.3						23.9		50.8		21.8					54.8		27.4		31.3				
**Lubl25**	**1995 [11]**	**human**	**Slovenia**	12	14	1284	905.5	27.3	60.4	21.3	24.5						24.1		50.8		28.4	8.2				54.9	21.2	27.8				30		
**PBae I**	**1990 [7]**	**human**	**Germany**	10	12	1251	905.4	27.1	60.5	21.4	24.9						32.5		46.8		28.3					54.9	21.2	27.8						
**PBae II**	**1990 [11]**	**human**	**Germany**	8	13	1139	894.8	26.9	60.4	20.7	25						23.9		49.2		26.5	11.3				*	*	*						
**PBar**	**1988 [6]**	**human**	**Germany**	12	14	1278	904.6	27.1	60.4	21.3	25.1						19.6		47.5		27.6	11.3				54.9	21.2	27.5				30		
**PBi**	**1984 [91]**	**human**	**Germany**	11	13	1280	905.8	27.1	60.4	21.2	24.6						24.1		50.9		22.8	*	28.4			54.9		28.6		31				
**PBN**	**1999 [7]**	**human**	**Germany**	11	13	1269	902.2	26.7	46.6	21.3	24.6						24.2		49.5				33.5			53.6		27.8		30.9				27.9
**PHer I**	**1989 [11]**	**human**	**Germany**	12	14	1287	905.2	27.1	60.3	21.5	24.9						24.2		50.6		27.7	12				54.8	21	27.8				30		
**PLad**	**2000 [11]**	**human**	**Germany**	12	14	1293	904.9	27.1	60.3	21.3	24.6						24.2		51		22.7	13.6	28.4			54.9		27.5		31.1				
**PNeb**	**1988 [7]**	**human**	**Germany**	12	14	1289	905.6	27.2	60.6	21.5	25.1						24.3		50.7		28.4	11.7				54.9	21.2	27.8				30		
**PNi**	**2000 [6]**	**human**	**Germany**	10	12	1240	905.7	27	46.6	21.3	24.6						24.1		48.8				28.4			55		26.9		31.1				
**PRab**	**1994 [7]**	**human**	**Austria**	11	13	1255	904.5	27	59.8	21.1	24.4						24		50.5		27.8	12				54.9	21.2	27.4						
**PRof**	**1989 [6]**	**human**	**Germany**	12	14	1288	903.8	27	60.4	21.4	24.6						24.1		50.4		22.6	13.3	26			54.9		27.2		31.1				
**PTrob**	**1988 [7]**	**human**	**Slovenia**	12	14	1290	905.5	27.2	60.5	21.4	24.4						24.1		51.1		28.2	12.9				54.9	21.2	28				30		
**PWin**	**1987 [6]**	**human**	**Germany**	12	14	1284	901.6	27.1	60.4	21.1	26						24.1		50.4		27.4	11.3				54.4	21.1	28.6				29.8		
**PZwi**	**1994 [6]**	**human**	**Germany**	11	13	1273	904.9	27.2	60.4	21.3	24.3						24.2		48.9		28.2					54.3	21	26.7				29.5		

The length of the main chromosome (chrom) and each assembled plasmid (of at least 5kb length) is shown. The three strains sequenced with both PacBio and Illumina data are shaded in gray. Date is date of isolation and is followed by the original reference (see Bibliography, if blank: this study). #PF32 is the number of Pfam 32 proteins identified by BLAST (see Methods) in the assembled data and in the contigs and * means a plasmid partition gene of the families (PF32, PF49, PF50 or PF57-62) was identified but no plasmid of at least 5kb length could be assembled. Length are in kb. °: isolate Konnai17 was a mixture of *B. afzelii* and *B. bavariensis*, we used clone number 1 which is only *B. bavariensis*

## Results

### *Borrelia bavariensis* genome reconstruction from next-generation sequencing data

The assembly of *B. burgdorferi s*.l. genomes is known to be difficult due to the fragmentation of the genome and to the presence of highly similar plasmids (like the cp32 plasmid family) [18]⁠. We used a combination of long read (PacBio) and short read (Illumina HiSeq and MiSeq) to overcome this problem.

For three isolates (the *B. bavariensis* type strain PBi from Germany, a second European isolate A104S from the Netherlands and the Japanese isolate NT24: highlighted in gray in Table 1), we used both sequencing techniques. For each isolate an assembly was first reconstructed using PacBio reads and then assembled contigs of Illumina short reads were mapped to the PacBio assemblies (see Methods). For most of the three genomes, the two methods gave very similar results with over 99.99% similarity between the Illumina contigs and the PacBio assemblies. Most differences were point mutations and 1 bp-long indels which are known to occur due to the lower accuracy of the PacBio sequencing method [30]⁠. In such cases, the Illumina version of the sequence was kept.

In one case, the Illumina data allowed us to correct a PacBio assembly. The PacBio assembly for isolate NT24 showed two plasmids of respective sizes of 107,820 bp and 49,218 bp that we originally named plasmids cp32–12 + 5 + 6 and cp32–7 + 7 + 11 due to the presence of the corresponding PFam32 sequences. These two plasmids seemed to be fusions of three cp32 plasmids each. Mapping the Illumina raw reads on these sequences (Suppl. Fig. 1) showed that several regions of those PacBio plasmids were not covered by Illumina reads which was not the case for other plasmids. The fusions were thus not supported and were probably an artifact of the PacBio assembly. We used contigs from other isolates as a reference for reconstructing the probable plasmids of the cp32 family in this isolate (see below).

In isolate PBi, unmapped Illumina reads contained sequences similar to lp28–8. This plasmid was not reconstructed in the PacBio assembly. Mapping of PBi Illumina contigs on A104S lp28–8 showed that five contigs mapped to this plasmid with 92–99% similarity to the A104S version. However, the original architecture of the plasmid in PBi was probably different as the five mapping contigs did not cover the full A104S sequence and were themselves not mapped over their whole length. Therefore, the final lp28–8 PBi plasmid sequence could not be reconstructed and, additionally, no PFam32 plasmid partition protein could be found for this plasmid. However, another plasmid partition protein of the family PFam50 for lp28–8 was identified in PBi showing that this plasmid is probably present.

For the remaining 30 isolates which were sequenced with Illumina only, we mapped contigs assembled with SPAdes v. 3.10.1 [31]⁠ to the final genomes of the three isolates sequenced with PacBio, as well as to plasmids identified as one full contig in the isolates sequenced with Illumina only, with NUCmer v. 3.1 from package MUMmer [32]⁠ (see Methods). Plasmid sequences were kept in the final reconstructed genomes only if they were at least 5000 bp long and were named after the PFam32 protein types identified in their sequence using BLAST v. 2.8.1 [33, 34]⁠ or after the reference they were mapped to in case of the absence of a PFam32 sequence (see Methods and Suppl. Table 1). To ensure that the assembly method chosen was good (SPAdes v. 3.10.1 [31]⁠⁠), we also assembled sequence data of 25 isolates with SOAPdenovo v. 1.0 [35]⁠ and VelevetOptimizer v. 1.0 [36]⁠ (see Methods) and used QUAST v. 4.6 [37]⁠⁠ to compare the quality of the three assemblies. As is shown in Supplementary Figure 2, N50 values were significantly higher in SPAdes assemblies compared to assemblies of the two other assemblers (Wilcoxon Rank Sum Tests with each other assembler: Bonferroni-Holm corrected *P*-Value < 0.01) and the number of contigs was significantly smaller (Wilcoxon Rank Sum Tests with each other assembler: Bonferroni-Holm corrected *P*-Value < 10^− 4^). In addition, the total length of the final assembly was largest in SPAdes in 24 out of 25 isolates tested. We conclude that, of the three tested assemblers, SPAdes performed the best.

We also remapped the raw Illumina reads on the final reconstructed genomes to check the quality of our reconstruction (see Methods) and show the relative standard deviation (SD) of coverage as a measure of quality in Supplementary Figure 3. A well assembled genome should have a low coverage variance as reads would map evenly to the contigs. The isolates from Asia showed a significantly higher variance in coverage (Wilcoxon Rank Sum Test: *P*-Value < 10^− 16^) as compared to the European isolates. This could be due to variation in the quality of the original DNA samples, (DNA samples from the Asian isolates were shipped to Germany), or to the lack of good references for certain plasmids due to the higher diversity observed in the Asian isolates. Indeed, the relative SD was higher for plasmids compared to the main chromosome in Asian isolates even if this difference was not significant (Suppl. Fig. 3b). The quality of the assembly did not depend on the method used for obtaining the final plasmid sequence (either as an own entire contig or with contigs mapped to a reference) (Suppl. Fig. 3a).

### Genome composition of 33 *B. bavariensis* isolates

The genomes of the 33 isolates consisted of a main chromosome and a variable number of plasmids (Table 1). Chromosomes were about 900 kb in size (size of reconstructed chromosome varied between 894,779 bp in isolate PBaeII and 906,948 bp in isolate NT24) and made up on average 72.1% of the total assembled genome. Eight to 18 individual plasmid sequences of at least 5000 bp could be assembled per isolate. Additional plasmid sequences were identified in 11 isolates due to the presence of partition genes or as some contigs mapped to plasmids identified in other isolates (Suppl. Table 1). However, these additional plasmids could not be fully assembled or the assembled sequence did not reach the 5000 bp criterion. Several reconstructed plasmid sequences, particularly of the lp28 and cp32 plasmid families, are very short (below 10 kb). It is probable that the sequence reconstructed here for these plasmids does not recover the full plasmid length and that the missing sequences were probably erroneously assembled in other contigs due to similarity. This confirms that short read sequencing alone is not sufficient to reconstruct plasmids from these families. Using long-read sequencing was very helpful in the assembly of plasmids in isolates PBi and A104S. However, even the PacBio assembly pipeline failed to reconstruct properly the cp32 content of isolate NT24. For this isolate we used the same strategy as for the isolates with only Illumina data (see Methods) and mapped Illumina contigs to cp32 plasmids from other isolates. This allowed us to reconstruct plasmids cp32–11 and cp32–12. For plasmids cp32–5, − 6 and − 7 no mapping was possible; we could only use Illumina contigs that were 7.3, 7.3 and 9.9 kb long, respectively, and probably do not represent the full plasmid (Table 1).

**Fig. 1:**
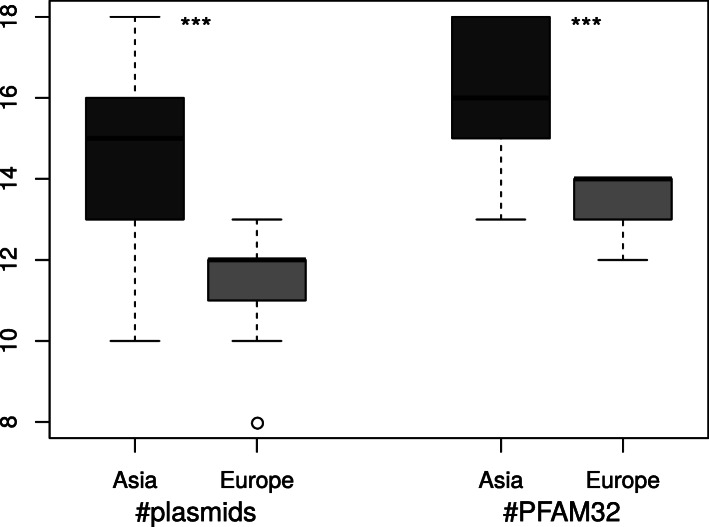
Asian isolates have more plasmids on average. Boxplots showing the number of plasmids and number of PFam32 proteins identified in the genomes of *B. bavariensis* isolates from Asia (dark grey) and Europe (light grey). ***: Wilcoxon Rank Sum test, *P*-value < 0.001

The number of plasmids per isolate (Fig. 1) was significantly higher in the Asian population (ranging from 10 to 18 reconstructed plasmids over 5 kb long) as compared to the European population (8 to 13 plasmids). As some plasmid fusions were observed and as some plasmids could not be reconstructed, we also tested for the number of PFam32 gene sequences present in each isolate. This was found to be significantly higher in Asian isolates compared to European isolates (Fig. 1), again implying that fewer plasmids are present in European isolates than in Asian isolates.

We also tested for a deviation in copy number between plasmids with respect to the main chromosome by plotting the coverage of the raw read mapping to each plasmid relative to the chromosome (Suppl. Fig. 4). As the coverage of Asian *B. bavariensis* genomes was more variable, we did this for European isolates only. We found the coverage of plasmids lp17, lp28–7 and lp36 to be significantly higher than for the main chromosome for all European isolates. In particular, based on this coverage measure, there were, on average, about seven copies of lp17 per cell in European isolates.

**Fig. 2:**
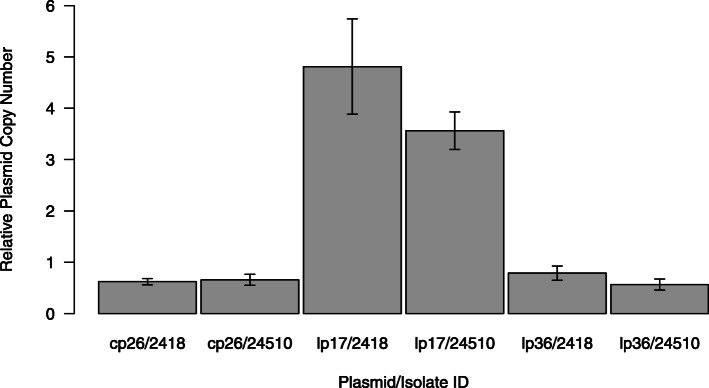
Relative plasmid copy number based on qPCR results. Relative plasmid copy number was estimated based on qPCR results on the chromosome and plasmids cp26, lp17 and lp36 on PBi isolates 2418 and 24510 ran with three biological and three technical replicates. Error bars represent the standard error of the mean

As several plasmids seemed to have a higher copy number compared to the main chromosome based on the read coverage of the Illumina data, we used a qPCR protocol to directly measure the number of DNA molecules present in a strain relative to the main chromosome. We chose to use plasmids cp26 (which we hypothesized to be present in about the same number as the main chromosome, based on read coverage) and lp17 and lp36 (which seemed to have higher copy numbers). We designed a qPCR protocol following Millan et al. [38]⁠ with one PCR per plasmid (see Methods for details) on two low passage isolates of *B. bavariensis* isolate PBi. Each isolate was run using three biological and two technical replicates. As can be seen in Fig. 2, the copy number of plasmid cp26 was estimated to be slightly below one copy per chromosome, that of lp36 was about one copy per chromosome and lp17 plasmid was found to be at a higher relative copy number varying between three and five copies per chromosome. This value is lower than the copy number estimated based on the coverage measure but is probably a more accurate estimate.

### Shared versus variable genome components

All *B. bavariensis* isolates sequenced in this study contained, in addition to the main chromosome, plasmids cp26, lp54, lp36, lp17 and lp28–4 (Table 1). In addition, we found in each isolate between 4 and 9 types of cp32 sequences. These were either fused with other plasmids or independent plasmids and their numbers were obtained by counting cp32 PFam32 sequences (as cp32 family plasmids could not be properly assembled in several isolates). Three cases of plasmid fusions were observed in at least two isolates and were thus considered to be true (other cases were not reported as they may have been due to mis-assembly and, in such cases, the plasmids were recorded without the possible fusion). In all European isolates, we observed two cases of fusion of a linear plasmid (lp28–4 or lp25) with a cp32 plasmid (cp32–1 and cp32–3, respectively). These fusions were found to be fixed in European *B. bavariensis* isolates but were absent from Asian isolates. In addition, plasmid lp17 and lp28–4 were found to be fused in four Asian isolates, but not in any of the European isolates. Interestingly, these isolates were found in independent clades in the phylogeny of the species (see below).

Supplementary Figure 5 shows a schematic representation of the fusions involving plasmids lp28–4, lp17 and cp32–1 with a precise description of the different plasmid types as well as plasmid lp28–7 as we found that translocations occurred within the European population between lp28–7 and lp17. To produce Supplementary Figure 5, we first had to determine plasmid types for the four plasmids under study. Following Casjens et al. [14]⁠ we counted a new plasmid type each time a deletion or insertion of at least 400 bp was observed and for each translocation or inversion of at least 400 bp (see Methods for more details). We were able to identify nine lp28–7 types, 12 lp17 types including two fusions with lp28–4, five other lp28–4 types, six cp32–1 types and six versions of the fused plasmid lp28–4 + cp32–1. There was no case of two Asian isolates sharing the same plasmid types for each one of these four plasmids (i.e. lp17; lp28–4; lp28–7 and cp32.-1) and in the European population we could identify only three groups of two isolates and one group of three isolates that shared the same plasmid types for lp28–7, lp17 and lp28–4 + cp32–1. Even if many short indels were observed on plasmid lp28–4, we could identify an almost 20 kb-long sequence that is shared by all types with or without fusions. The fusion of plasmids lp17 and lp28–4 in four Asian isolates was found to have occurred without any other big rearrangements. However, we identified two different architectures for this fusion. In isolate J-14 (and in isolates FujiP2 and Hiratsuka that were mapped to it) we observed a fusion of the 5′ ends of plasmids lp17 and lp28–4, thus lp17 appeared to be flipped. In isolate Arh923, the two plasmids were fused by their 3′ ends. Of course, this could have been due to mis-assembly. The fusion of lp28–4 and cp32–1, that is fixed in the European population, was shown to be an insertion of cp32–1 into lp28–4. There were two very different types of cp32–1 plasmids in the Asian population, with only about 10 kb homology. The fused plasmid observed in the European isolates seems to have occurred using the cp32–1 type carried by Asian isolate Hiratsuka (or a related cp32–1 type), which does not have more than 2 kb homology with the other Asian type of cp32–1. Apart from these two fusions, we could also observe a reciprocal translocation that occurred between plasmids lp28–7 and lp17 in the European population. Five European isolates including the reference strain PBi carry at the end of plasmid lp17 a 2.5 kb-long sequence that is found at the beginning of plasmid lp28–7 in all other isolates. And reciprocally, plasmid lp28–7 of three of these five isolates (the other two do not have a lp28–7) carry at their beginning a 5 kb-long sequence that is found at the end of lp17 in all other isolates. Both regions contained genes encoding outer membrane proteins.

**Fig. 3:**
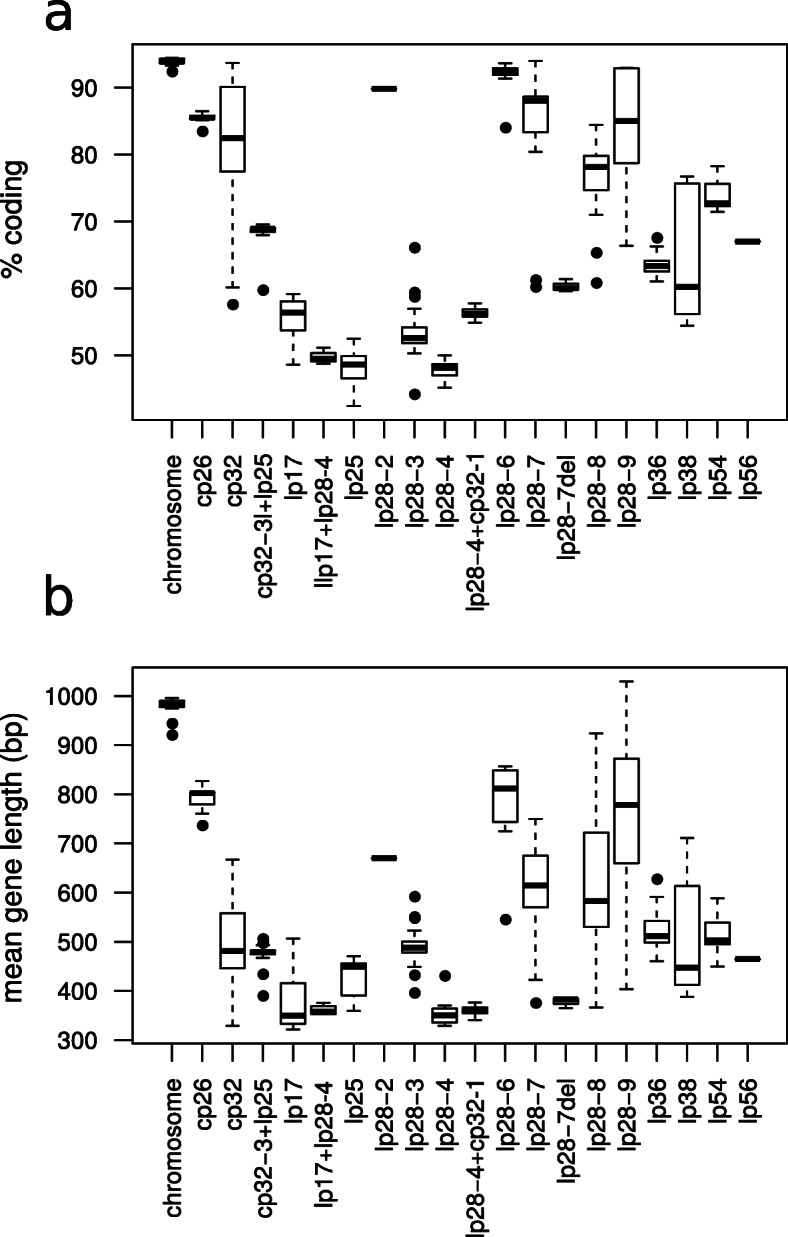
Gene content of the *B. bavariensis* replicons. Percentage of coding sequence (**a**) and average gene length (**b**) for the chromosome and each plasmid over isolates are shown as boxplots

We used RAST [39, 40]⁠ to annotate the reconstructed *B. bavariensis* genomes and, following the method by Mongodin and colleagues [17]⁠, kept all detected genes of at least 50 amino-acid length. The main chromosome was found to contain on average 816.4 genes that met this criterion (range 812–842) and on average 94% of the chromosome sequences were coding with very low variation among isolates (standard deviation 0.41 – see Fig. 3a). This was significantly higher than in plasmids (Welsh T test *T* = 32.0, df = 427, *P*-value < 0.001). Circular plasmids had a significantly higher percentage of coding sequence compared to linear plasmids (average circular: 82.4%, average linear: 66.1%, Welsh T test, *T* = 14.6, df = 366, *P*-value < 0.001; fusions between circular and linear plasmids were excluded). As shown in Fig. 3b, annotated genes were also significantly longer on the chromosome compared to the plasmids (mean 981 bp, Welsh T test *T* = 65.0, df = 434, *P*-value < 0.001). Circular plasmids had significantly longer genes compared to linear plasmids (average circular: 562 bp, average linear: 514 bp, Welsh T test, *T* = 3.4, df = 326, *P*-value < 0.001; fusions between circular and linear plasmids were excluded). We used BLAST v. 2.8.1 [33, 34]⁠ at the amino-acid level (algorithm BLASTp) to compare each of the 33 isolates with the 32 others for gene content. A hit between protein sequences in two different isolates was kept if the hit had at least half the length of the original gene and if the identity between the two sequences was as least 90%. Using these criteria, we found that at least 93% of the genes located on each chromosome had a hit on every other chromosome. This confirmed that the chromosome was highly conserved within the species *B. bavariensis,* even between Asian and European isolates. Indeed the best hits between isolates for each chromosomal gene had on average 98.8% sequence identity when the compared genes were from isolates from within the same continent and 97.5% when the compared isolates were from different continents. Plasmid cp26 was also found to be highly conserved with on average 91.1% of its 26 to 28 genes being shared with the cp26 plasmids of each other isolate and the identity of the best hit in each isolate being on average 99.0% for isolates from the same continent and 92.7% for isolates from the other continent.

Out of the 24 different plasmids assembled from the genomic data of the 33 *B. bavariensis* isolates (without taking fusions into account), 19 were not found in all isolates. This estimated variable portion represented on average 19.2% of the total reconstructed genomic content of each isolate and 68.3% of the total assembled plasmid content. These size estimates of the variable genome represent only a lower bound because some plasmids found in all isolates are nevertheless not similar over their whole length and some plasmids were not successfully assembled. The greatest degree of diversity was observed on the two plasmid families lp28 and cp32 which were represented by seven and ten members, respectively, over all isolates with only lp28–4 found to be present in every isolate.

### Evolution of the species

**Fig. 4:**
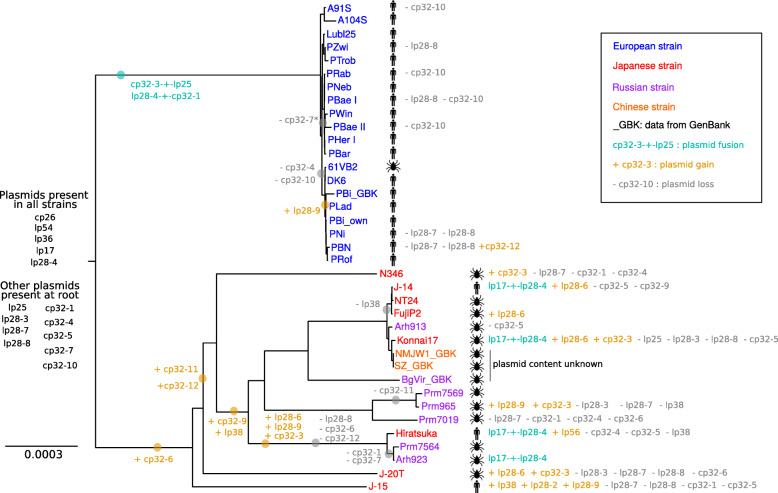
Phylogeny of *B. bavariensis* reconstructed based on the main chromosome. Phylogeny reconstructed with BEAST v1.8.0 [41]⁠ with the following parameters: coalescent model with exponential growth based on doubling time, lognormal-relaxed clock [42]⁠, GTR substitution model [43]⁠. A burn-in of 30% of the 100 Million steps chain was removed before selecting the best tree with TreeAnnotator v. 1.10.4 [41]⁠. The scale is in substitutions per site. Node posterior probabilities were above 0.99 for all nodes except in the European clade (very short branches and very low diversity). The gain (orange), loss (gray) and fusion (cyan) events were positioned following maximum parsimony principle. There are indicated on a branch if they concern several isolates and after the isolate name if they concern only one isolate. Isolate origin is indicated by a tick for isolation from a tick (*I. persulcatus* in Asia and species unknown for European isolate 61VB2) and a human for isolation from a human patient. The accession numbers for the sequences coming from public databases can be found in the Methods section. * This plasmid loss event concerns the branch leading to isolates Lubl25, PZwi, PTrob, PRab, PNeb, PBae I, PWin, PBae II, PHer I and PBar

We used BEAST v1.8.0 [41]⁠ to reconstruct the phylogeny of the main chromosome (see Methods for more details) for all of our 33 isolates as well as four additional isolates for which chromosomal sequences have been published in GenBank (under accession numbers CP000013 for strain PBi from Germany, CP003151 for strain BgVir from Russia and CP003866 and CP007564 for strains NMJW1 and SZ from China). We used *B. garinii* strain 20047 as an outgroup to root the tree (GenBank accession number CP028861). The resulting phylogeny, presented in Fig. 4, shows that the two continental populations are clearly divergent with a deep branching. The European population is characterized by a very short-time divergence and an almost clonal recent evolution as has already been noted [11]⁠. The Asian population, even if showing greater overall divergence, does not show any geographical structure: isolates from Japan, China and Russia are found in the same terminal clades. Asian isolates also did not cluster by origin of the isolate (questing tick or patient). In Europe, only one isolate from a tick was available and this had no special position in the phylogeny. Both chromosome assemblies for the PBi type strain (ours and that published as CP000013) were both located in the same clade. We compared RAST [39, 40]⁠ annotation results for both PBi chromosome sequences and found that there was perfect synteny between the two (Suppl. Fig. 6).

In this phylogeny, we also indicated gains, losses and fusions of plasmids based on the reconstructed genomes using maximum parsimony (Table 1 and Suppl. Table 1). This showed that, in addition to five plasmids present in all isolates, four other linear plasmids and five cp32 plasmids could have been present at the root of the tree in the ancestral *B. bavariensis.* These plasmids would then have been subsequently lost in some derived isolates. Nine gain and ten loss events could be placed on internal branches and thus were shared by at least two isolates. In the European clade, three plasmid loss events on internal branches and ten plasmid loss event on terminal branches were found, whereas only two gain events were identified (plasmid lp28–9 shared by five isolates and plasmid cp32–12 found only in isolate PBN). This shows that the plasmid repertoire of the European population is rather stable with only plasmid losses that could have been due to isolate cultivation in the laboratory rather than to real evolutionary change. In the Asian population, according to our maximum parsimony reconstruction, plasmid gains were as frequent as plasmid losses on internal branches (eight gain events for seven loss events) but there were twice as many losses as gains on terminal branches (13 gains for 29 losses).

Genetic diversity within and between the Asian and European populations was estimated by nucleotide diversity (*π* [44]⁠) and genetic distance (*F*_*ST*_ [45]⁠) for the main chromosome and seven plasmids with orthologous regions in at least five isolates in each population (see Methods, Table 2). Diversity was found to be lower in the European population compared to the Asian population by one to two orders of magnitude depending on the genomic segment and to be lower for the main chromosome compared to plasmids. Genetic distance between Asian and European populations was lowest for lp25 (0.36) and highest on lp36 (0.69).

**Table 2:** Within and between population genetic diversity for the main chromosome and plasmid orthologous regions

Genomic region	# Asia	# Europe	Length (bp)	# SNP	*π Asia*	*π* Europe	*F*_*ST*_
chromosome	17	19	920,528	42,039	8.79*10^− 3^	1.72*10^− 4^	0.56
cp26	15	19	29,623	1979	1.54*10^−2^	1.99*10^−4^	0.50
lp17	14	19	13,732	1331	1.98*10^−2^	4.99*10^−4^	0.49
lp25	13	18	27,833	3232	2.97*10^−2^	7.03*10^−4^	0.36
lp28-3	11	19	11,152	1572	6.80*10^−2^	8.23*10^−4^	0.50
lp28-4	14	18	31,849	4144	2.05*10^−2^	2.62*10^−3^	0.52
lp36	14	19	9819	1081	2.34*10^−2^	3.66*10^−4^	0.69
lp54	15	19	67,261	8167	2.06*10^−2^	3.47*10^−4^	0.59

Genetic diversity (*π* [44]⁠) within populations and genetic distance (*F*_*ST*_ [45]⁠) between populations were estimated on orthologous sequences aligned with MAFFT v 7.407 [46, 47]⁠. The number of single nucleotide polymorphisms (SNP) is indicated for both populations mixed and the length is the length of the alignment

We also estimated genetic diversity along the main chromosome and for plasmids cp26 and lp54, in which alignments were possible over the whole length (Suppl. Figs. 7, 8 and 9). For all three replicons, we identified peaks of diversity either between populations from the two continents (peak only when considering all isolates) or in one or both regional populations. We found high diversity in several chromosomal genes coding for proteins located in the outer membrane of the bacteria (OppA, ABC transporter, Lmp1, PTS system). This was also true for lp54, particularly in the Asian population, with diversity peaks located in the genes encoding OspA, OspB, DbpA and in the PFam54 gene array. On cp26, the o*spC* gene is well known for having high diversity in several *B. burgdorferi* s.l. species including *B. bavariensis* which is confirmed here for the Asian population [11, 17, 48, 49]⁠.

**Fig. 5:**
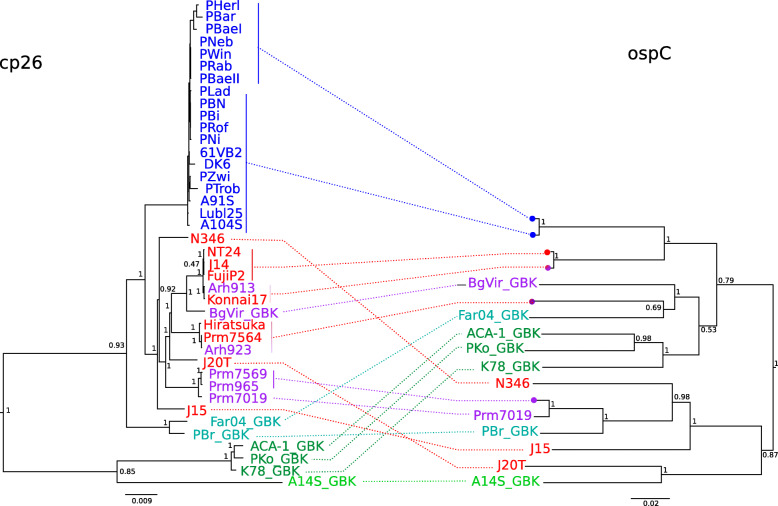
Comparison of cp26 and *ospC* phylogenies. Sequences for the *ospC* gene and the cp26 plasmid without *ospC* (cutting out 200 bp upstream and downstream the gene) were aligned with MAFFT v7.407 [46, 47]⁠ and BEAST v1.8.0 [41]⁠ was run for 100 Million states for cp26 and 20 Million states for *ospC* each in triplicate. Best trees were reconstructed after removing a burnin-in of 10% of the chain and all three runs showed very similar results for each tree. Both trees were plotted using FigTree v. 1.4.4 (http://tree.bio.ed.ac.uk/software/figtree/) and manually rotated. Color code for isolates: Light green: *B. spielmanii*, dark green: *B. afzelii*, cyan: *B. garinii*, purple: *B. bavariensis* Russia, red: *B. bavariensis* Japan, marine blue: *B. bavariensis* Europe. Dots on the *ospC* phylogeny represent several isolates having exactly the same sequence. Scale bars are in substitutions per site. Values next to nodes indicate node posterior probability (not shown within the European *B. bavariensis* clade for the sake of clarity)

As *ospC* showed a high diversity, and as this locus is known to be a hotspot of recombination in several *B. burgdorferi* s.l. species [11, 48, 50]⁠, we reconstructed a phylogeny of this gene and compared it to that of the cp26 plasmid cutting out the *ospC* locus. Several publicly available sequences for *B. bavariensis* (strain BgVir), *B. garinii* (strains Far04 and PBr), *B. afzelii* (strains ACA-1, K78 and PKo) and *B. spielmanii* (strain A14S) (see Methods for details) were additionally included in this analysis. As can be seen in Fig. 5, the cp26 phylogeny followed the known species tree with *B. bavariensis* and *B. garinii* being sister species as are *B. afzelii* and *B. spielmanii*. The phylogeny of plasmid cp26 within *B. bavariensis* was very similar to the phylogeny reconstructed for the main chromosome (Fig. 4), except for minor differences in clustering of Japanese isolates. However, the phylogeny reconstructed for *ospC* was quite different and showed two major clades. One clade contained all European *B. bavariensis* and all *B. afzelii* as well as some Asian *B. bavariensis* and one of the two *B. garinii* strains. The second clade contained *B. spielmanii*, the other *B. garinii* strain and the rest of the Asian *B. bavariensis* haplotypes. Apart from the European *B. bavariensis* clade (where we observed only two different *ospC* haplotypes with only one non-synonymous difference between them) and the *B. afzelii* clade, all other species or populations with several isolates were found not to be monophyletic.

## Discussion

### Strategies for genome reconstruction of *B. burgdorferi* sensu lato

In this article, we present genome reconstructions for 33 *B. bavariensis* isolates from Eurasia. Following other studies (see for example [18]⁠⁠), we used a combination of long-read (Pacific Bioscience) and short-read (Illumina) sequencing. We show that the PacBio long-read assembly allowed the reconstruction of most plasmids even from the cp32 and lp28 families. It had been reported before that PacBio assemblies contain inaccuracies [51]⁠ and in one out of the three isolates, the PacBio assembly created two, probably spurious, fusions of plasmids belonging to the cp32 family. This occurred in one Japanese isolate that possessed nine cp32 plasmids, the maximum of cp32s observed in our sample set. It shows that proper assembly of sequences carrying so many cp32 plasmids remains challenging even when using long-read data. However, fusions of cp32 plasmids have been observed in other species of the *B. burgdorferi* s.l. complex [52, 53]⁠ and it remains an unresolved question whether these were real in isolate NT24. In isolate PBi, Illumina reads were identified that mapped to plasmid lp28–8 and carried the lp28–8 PFam32 sequence but no contigs for this plasmid were found in the PacBio assembly. The Illumina data for this plasmid was too fragmented to reconstruct the plasmid sequence via mapping. Thus, it is possible that this plasmid was not present in each cell of the isolate or was in the process of decaying or being lost while cultivating the isolate for DNA extraction as has been described in many *Borrelia burgdorferi* s.l. isolates [54–56]⁠. Although circular consensus sequencing (CCS) improved the accuracy of PacBio data, it has been established that long-read data is more prone to sequencing errors [30]⁠. It is therefore advisable to complement and correct them using more accurate short-read data. Reassuringly, for each replicon, the similarity between PacBio and Illumina reads was above 99.98%.

For the 30 isolates for which no long-read sequencing data was available, our strategy was to perform de novo assembly of the Illumina reads and then use the three long-read isolates as a reference for mapping if required. For some replicons, the mapping step was not necessary as single contigs were available that covered whole plasmids. This was the case for five out of 30 chromosomes and for numerous plasmids (as an example, all but five cp26 plasmids were each covered by a single contig). It made no noticeable difference for assembly accuracy (Suppl. Fig. 3), whether the data was mapped or assembled directly as one contig. Such contigs that assembled as full plasmids were successfully used as references for other isolates. Despite all this, for 11 isolates, a total of 27 plasmids were missing from, or incomplete in, the final assembly. These replicons were known to be present as plasmid partition gene sequences for them were identified or as contigs mapped to them, but we could not reconstruct a full plasmid. Perhaps not surprising, this happened more frequently in the Asian isolates (in nine isolates a total of 23 plasmids were missing) than in the European isolates (two isolates and four plasmids). Whether this was due to a lower data quality in the Asian isolates and/or challenges to find an appropriate reference (due to the higher diversity in plasmid content observed in this population) is currently unclear. In addition, several reconstructed plasmids were very short and it is probable that part of their sequence was not assembled.

The use of only short-read sequencing thus resulted in a good global description of the plasmid content, but proper full genome reconstruction was only possible in those isolates for which a close reference was available, as was the case for the European isolates. This was also the case in previous studies using Illumina short-read sequencing in *B. burgdorferi* s.s. (see for example [57]⁠).

### The *B. bavariensis* genome shows a high degree of conservation

The core genome of the species complex *B. burgdorferi* s.l. is considered to be composed of the main chromosome and plasmids cp26 and lp54 [17]⁠. In addition, all the *B. bavariensis* isolates sequenced here share sequence stretches of three other plasmids: lp17, lp28–4 and lp36. Interestingly, 14 strains of *B. burgdorferi* s.s. have also been shown to share these same five plasmids (cp26, lp17, lp28–4, lp36 and lp54) [14]⁠⁠. For plasmids lp17 and lp28–4, the shared sequence stretches made up about 12 kb and 18 kb, respectively, and for plasmid lp36 a fragment of about 13 kb was found to be shared among all isolates. These sequences can thus be considered as belonging to the core genome of *B. bavariensis* which thus adds up to 1027 kb; being made up of 900 kb of chromosomal sequence plus 127 kb of plasmid content (with 27 kb on cp26 and 57 kb on lp54). The chromosome and cp26 sequences are, in particular, highly conserved as seen when comparing gene content between isolates and as already described [14, 17]⁠⁠. A very high proportion of the genes on these two replicons (93% for the main chromosome and 91.1% for cp26) are found in all isolates.

The main chromosome sequences also allowed us to reconstruct a phylogeny for the species (Fig. 4). We had already published a similar phylogeny using a subset of these isolates [11]⁠. However, the Russian isolates are new to the present paper and allow us to see that the Asian clade shows no detectable geographic clustering. Asian *B. bavariensis* are vectored by *I. persulcatus*, whereas the European vector is *I. ricinus* (see [9]⁠ for a review). As these two tick species co-occur and can even hybridize in their overlapping zone in Estonia, Latvia and Western Russia [58]⁠, we expected that Russian *B. bavariensis* samples, might be genetically closer to the European isolates than the Japanese isolates, perhaps even showing that the European population might have diverged from a Russian lineage, but this was not the case. The lack of spatial structure in the Asian *B. bavariensis* genomes over such a large geographical scale can be explained either (i) by the co-occurrence over a long evolutionary period of many strains in the same populations due to specialization to some specific niches (like reservoir hosts) or (ii) by recurrent migration of strains, for example carried by ticks attached to birds. However, this last hypothesis seems less likely as *B. bavariensis* is rodent-adapted and does not survive in bird complement active immune serum [7, 59]⁠.

Another conserved pattern was the elevated coverage of the sequence data observed on certain plasmids and particularly on plasmid lp17 with respect to the main chromosome. The coverage of lp17 was higher than that of the chromosome in all isolates (European isolates are shown in Suppl. Fig. 4). This suggests that *B. bavariensis* normally carries a higher copy number of plasmid lp17 than is the case for other plasmids or the main chromosome. In another study, the coverage of a plasmid, lp28–6, in one *B. burgdorferi* s.s. strain was also found to be about ten times higher than then rest of the genome [25]⁠ but, to our knowledge, no study reported such a pattern for a plasmid in many isolates of the same species. We experimentally confirmed that the copy number of plasmid lp17 was three to five fold that of the main chromosome for isolate PBi grown under lab conditions (Fig. 2). This finding contradicts the current view of plasmid partitioning in *B. burgdorferi* s.l. according to which each plasmid is expected to contain at maximum one or two copies of each plasmid per cell [25, 60]⁠. The only other study we could find that experimentally tested for copy-number of plasmids in *B. burgdorferi* s.l. was performed on three plasmids of the *B. burgdorferi* s.s. reference strain B31 via relative hybridizations of replicon-specific DNA probes [61]⁠. These three plasmids were found to be present at about one copy per chromosome and this was shown to be stable when the strain was kept in culture. Outer membrane vesicles (OMVs) produced by *B. burgdorferi* s.l. bacteria could provide an explanation for DNA extracted from cultures possessing more copies of certain plasmids than the chromosome. OMVs are membrane-enclosed spheres that many bacteria, including *B. burgdorferi* s.l., fill with different molecules and release into their surroundings [62]⁠, often as a response to stress [63]⁠ that can be induced by cultivation conditions [64]⁠. OMVs produced by *B. burgdorferi* s.s. have been found to contain both circular and linear DNA [65]⁠. More recently, *B. burgdorferi* s.s. OMVs were also found to contain RNA preferentially transcribed from plasmid sequences but not specifically from lp17 [66]⁠. It is known from other bacterial species that such vesicles can be involved in toxin delivery, cell-cell signal trafficking, protein transfer, and horizontal gene transfer [67]⁠. Plasmids can be transferred via vesicles, and plasmid identity has been shown to strongly influence the efficiency of their loading into vesicles in *E. coli* [68]⁠. Taking all of this into account, together with the fact that lp17 has been shown to be involved in host tissue colonization and evasion of host immunity in *B. burgdorferi* s.s [69, 70].⁠, it is possible that *B. bavariensis* preferentially packages lp17 plasmids into OMVs and that these extra plasmid copies are the reason for the observed increased plasmid to chromosome coverage ratio in *B. bavariensis* isolates that were cultivated to high density, and thus under stressful conditions. This hypothesis, however, remains to be tested.

A further level of genetic conservation can be seen within populations and particularly in the European isolates. The genetic diversity on the chromosome and on plasmids is very low within the European population (Table 2) and even the ospC locus, which is known to be one of the loci with the highest within-population diversity on the *B. burgdorferi* sensu lato genomes [48, 49, 57, 71]⁠, shows very little variation in this population (Fig. 5). All the sequenced European isolates also share the presence of three plasmids (lp28–3, cp32–3 + lp25 and cp32–5) in addition to the 5 plasmids present in all *B. bavariensis* isolates. Two plasmid fusions are also shared by all European isolates. However, the European population is not as clonal as previously thought [6, 11, 72]⁠ and several plasmids have evidently been lost or gained during its evolution (Table 1 and Fig. 4). In contrast to the Asian population, the European population shows some degree of geographic structure, with the first node separating the two Dutch isolates (A104S and A91S), that are the most western isolates in our sample, from the rest of the population and with the two Slovenian isolates (Lubl25 and PTrob) also being in the same clade together with a German isolate (PZwi).

The Asian population showed more variability, both at the sequence level and in the plasmid repertoire (we could find no pair of Asian isolates having exactly the same plasmid content based on the distribution of the PFam32 sequences). All the Asian isolates are characterized by a higher number of plasmids compared to European isolates and in particular by a higher number of cp32 plasmids (7.3 on average against 4.6 for the European isolates). This large cp32 repertoire might be associated with the ability to infect a wider range of vertebrate hosts; in *B. burgdorferi* s.s. cp32 plasmids carry several genes essential for host infectivity among which are the loci coding for Erp proteins that have been shown to bind complement proteins in humans (see [73]⁠ for a review).

### The *B. bavariensis* genome also displays a high degree of plasticity

While part of the *B. bavariensis* genome was found to be highly conserved, we also observed a high diversity, in particular in plasmid content. About two thirds of the plasmid content of each isolate was not shared by the whole species. This has been observed in *B. burgdorferi* s.s. as well [18, 25]⁠. We placed gains and losses of plasmids on our *B. bavariensis* phylogeny based on the main chromosome using maximum parsimony (Fig. 4). According to this reconstruction, 14 out of 24 plasmids would have been present in the common ancestor of the species. It is important here to remind the reader that plasmid loss can occur while *B. burgdorferi* s.l. bacteria are grown in culture, and that this could be the reason for the absence of some plasmids from certain isolates [54–56]⁠. Thus, some of the apparent plasmid losses during *B. bavariensis* phylogeny may not be real. Nevertheless, it is very unlikely that all the apparent losses of plasmids are artifactual, and gains of plasmids cannot be explained in this way. The complexity of the evolution of plasmid content in *B. bavariensis*, as depicted in Fig. 4, is striking and shows that the plasmid fraction of the genome is very plastic as has also been shown for *B. burgdorferi* s.s. [14]. The ability to exchange plasmids, either via OMVs as described above or using other mechanisms, seems to be very pronounced in *B. bavariensis* and in particular in the Asian population.

The genome plasticity of *B. bavariensis* is further demonstrated by the occurrence of three plasmid fusions shared by at least two isolates. Two of these fusions are fixed in the European population and concern the fusion of a member of the cp32 family with a linear plasmid. Such a fusion between a linear plasmid and a cp32 plasmid has been previously observed in plasmid lp56 of *B. burgdorferi* s.s. type strain B31 [74]⁠. We identified one lp56 plasmid in the Japanese isolate Hiratsuka based on the PFam32 protein (86.56% identity to B31 PFam32 sequence for lp56). However, this probably incomplete plasmid was made only of one 23 kb-long contig and showed only very little sequence similarity with its counterpart in strain B31. The third fusion (lp17 + lp28–4) occurred in several Asian isolates and is not monophyletic in the phylogeny depicted in Fig. 4. It was thus probably inherited horizontally and, as it is present in two out of the three Asian isolates coming from patients, one may speculate that it is linked to specific virulence factors. The presence of two different versions of this fused plasmid that differ in the point of fusion (Suppl. Fig. 5) implies that plasmids lp17 and lp28–4 were involved in at least two different fusion or recombination events. Similar fusion or relocation events have been previously observed in other genospecies. Plasmid lp17, for example, has also been suggested to have been involved in multiple relocations and fusions in *B. burgdorferi* s.s. [14].

### Candidate genes for host and vector adaptation in *B. bavariensis*

Whereas the plasmid content in the European population was rather well conserved, plasmid lp28–9 was found only in a single European clade made up of five isolates (including the type strain PBi) and was absent from all other European *B. bavariensis* isolates. Plasmid lp28–9 was however present in five Asian isolates (two of which were isolated from patients) and in the two published strains from the sister species *B. garinii*. Annotation of this plasmid in the European isolates allowed us to identify only one gene with a predicted putative function: it is an ortholog of a lp28–2-located gene, BBG11, from *B. burgdorferi* s.s. strain 297 that has been shown to be upregulated in rodent hosts by the RpoS transcription factor [75]⁠ and to have higher expression levels in *B. burgdorferi* s.s. infecting steroid-treated non-human primates compared to immuno-competent animals [76]⁠. This gene was found to be present only on the lp28–9 from European isolates and on some, but not all, of the lp28–7 and lp28–6 plasmids of some Asian isolates. Further research is necessary to find the function of this gene and whether it plays a role in pathogenicity in humans.

Other interesting genes highlighted by our study are those located on genetic diversity peaks (Suppl. Figs. 7, 8 and 9) within or between the two *B. bavariensis* populations. Because all Asian *B. bavariensis* isolates are vectored by *I. persulcatus*, whereas European isolates are found only in *I. ricinus*, it has been hypothesized that it is the adaptation to a new vector species that caused the strong bottleneck observed in the European population (see [9]⁠ for a review). Genes that show a high differentiation between the two populations are particularly interesting candidates for playing a role in the adaptation to specific tick vector species. Good examples of such genes are those encoding OspA, OspB and OspC located on lp54 (OspA, OspB) and cp26 (OspC) that showed a high diversity in the Asian population but were not variable at the amino-acid level in the European population. These proteins are known to be involved in the interaction between *B. burgdorferi* s.l. bacteria and their vectors and hosts (see [77]⁠ for a review). Topological differences that are observed in phylogenies of *ospC* and the rest of cp26 (Fig. 5) implies that differential evolution processes acted on the *ospC* gene and on plasmid cp26 during *B. bavariensis* evolution. A similar discrepancy has also been shown at the level of the *B. burgdorferi* s.l. species complex [78]⁠.

OspA and OspB could possibly be associated with evasion of the tick immune system as both genes are known to be expressed during infection of the tick [79]⁠⁠. Genetic variation at the *ospA* locus has already been observed in *B. garinii* [80]⁠. This diversity, however, does not coincide with different vector use. Other species such as *B. afzelii* and *B. burgdorferi* s.s., which can also use different vectors, have a rather conserved *ospA* [80]⁠. It may be that OspA fulfills different functions within the tick apart from its role as receptor binding molecule to TROSPA [81]⁠.

Another peak of genetic diversity was observed between 52 and 60 kb of the aligned plasmid lp54. RAST annotation [39, 40]⁠ did not show genes with known function in this region but the end of lp54 is known to be the region encoding the PFam54 protein family [74]⁠. Indeed we were able to identify the genes encoding for CspA-related PFam54 proteins BGA66 and BGA71 on the region showing high diversity in both *B. bavariensis* populations (Suppl. Fig. 9). The CspA protein was first identified in *B. burgdorferi* s.s. and is known to be involved in the evasion of the innate immune response in the human host by binding regulators of the complement system [82, 83]⁠. CspA was later shown to belong to a large protein family (PFam54) that is known to be under fast adaptive evolution [84]⁠. Our results are in accordance with this finding. In particular, the evolution of the *B. bavariensis* PFam54 members BGA66 and BGA71 is of high interest as these proteins have been found to be involved in complement inactivation in *B. bavariensis* reference strain PBi but with a different mechanism as compared to the *B. burgdorferi* s. s. CspA protein [85]⁠.

## Conclusions

Reconstruction of almost complete genomes of 33 *B. bavariensis* isolates from Eurasia showed that this species is characterized by a high degree of genetic conservation combined with plasticity. Asian isolates were found to have a high diversity in plasmid content and showed no geographic structuring. The European population was less diverse, appearing to have undergone a genetic bottleneck, but still showed some heterogeneous plasmid content. Two plasmid fusions were fixed in the latter population with respect to the Asian population. Horizontal transfer of genes or whole plasmids and gain and loss of plasmids likely influenced the evolution of this species. This study opens the way to functional genomic research on genes that have specific evolution pattern in this species and are thus good candidates for vector and host adaptation and for human pathogenicity.

## Methods

### Isolates used and sequencing

Information on origin of the isolates used for this study can be found in Table 1. All the European isolates from the strain bank of the German National Reference Center for *Borrelia* at the Bavarian Health and Food Safety Authority (Bayerisches Landesamt für Gesundheit und Lebensmittelsicherheit). Seventeen isolates were isolated from patients and one isolate was from a questing tick. The Asian isolates were isolated from questing ticks or patients in Russia and Japan.

*Borrelia bavariensis* were cultured in inhouse-made MKP (European samples) or inhouse-made BSK (Russian and Japanese samples) medium using standard procedures [86]⁠ to density of at least 10^8^ cells per *mL* in order to obtain enough DNA. DNA was extracted using a Maxwell® 16 LED DNA kit (Promega, Germany) and Japanese isolates were purified using Wizard genomic DNA purification kit (Promega). DNA concentrations and quality (260/280) were determined by using a Qubit® fluorometer 3.0 and (Thermo Fisher Scientific, USA) and NanoDrop® 1000 photometer (Thermo Fisher Scientific, USA).

For all 33 isolates, libraries were prepared according to the Nextera DNA sample preparation guide (Illumina, San Diego CA, USA). The samples were diluted to a DNA concentration of 0.2 ng/μl and “tagmented” by simultaneously fragmenting DNA using transposomes as provided by the manufacturer and adding adapters. After tagmentation, samples having adapters on both ends underwent five PCR cycles to amplify the product and to add index primers. The resulting libraries were then validated using an Agilent 2100 Bioanalyzer (Agilent, Germany). We then sequenced using an Illumina MiSeq platform (Illumina, San Diego CA, USA) that produced paired-end reads of 250 bp. Some low quality samples (A104S, DK6, PBae I, PBae II, PBar, PBN, PLad, PWin and PZwi) were repeated on an Illumina HiSeq platform producing 100 bp long paired-end reads.

For isolates PBi, A104S and NT24, Pacific Bioscience SMRT sequencing (hereafter PacBio) was performed using 10 μg of DNA. A library was prepared using Pacific Biosciences 20 kb library preparation protocol. Size selection of the final library was performed using BluePippin with a 10 kb cut-off. The library was sequenced on a Pacific Biosciences RS II instrument using P6-C4 chemistry with 360 min movie time.

### Genome assembly and mapping

PacBio reads were assembled using HGAP v3 (Pacific Biosciences, SMRT Analysis Software v2.3.0). Chromosomes and linear plasmids 3′ and 5′ ends were trimmed for removing the pseudo-telomere regions that are known to be present in *B. burgdorferi* s.l. linear replicons [87]⁠. Illumina contigs (see below) for the same isolates were then mapped to the PacBio assembly with NUCmer v. 3.1 from package MUMmer [32]⁠. As PacBio sequencing technology is prone to sequencing errors like point mutations and short indels [30]⁠, we combined the data from PacBio and Illumina using the following rules: for each indel of length 5 bp or less keep the Illumina version, for longer indels keep the PacBio version. For point mutations, keep the Illumina version if all contigs mapping on this position agree, else keep PacBio version.

Illumina reads were assembled using SPAdes v. 3.10.1 [31]⁠. As a comparison, we also assembled 25 isolates with SOAPdenovo v. 1.0 [35]⁠ and VelevetOptimizer v. 1.0 [36]⁠ and used QUAST v. 4.6 [37]⁠ to compare the quality of the three assemblies.

Mapping of SPAdes contigs was performed with NUCmer v. 3.1 from package MUMmer [32]⁠ on each one of the three isolates sequenced with PacBio that were used as reference. Contigs that were identified as being a whole chromosome (five cases) or a whole plasmid were used as is. For sequences that needed mapping of several contigs, the closest reference was used (highest identity and longest sequence reconstructed). This reference could be from one of the three PacBio isolates but also a contig identified as a whole plasmid in another Illumina isolate (61VB2 lp17, lp28–8 and cp32–5, A91S lp36, Arh923 lp28–7, FujiP2 lp28–6, Hiratsuka cp32–9 and cp32–11, J-14 lp17, lp28–4, lp28–6, cp32–7, cp32–10, cp32–11 and cp32–12, J-20 T lp25 and cp32–4, Lubl25 lp28–7, PBae II lp28–8, PBar lp54 and lp28–4 + cp32–1, PBN lp28–3, PHer I lp36, PLad lp28–8, PNeb lp36, lp17, lp28–7 and lp28–8, Prm7019 lp28–8 andPrm7569 cp32–1 were used as reference for other isolates). Each mapping file was then curated to suppress contigs overlapping other ones with higher identity (often these were very short contigs that mapped with low identity to a region already covered by a longer contig). We also corrected cases where one contig was supposed to map to several plasmids (often from the lp28 or cp32 families) or contigs which did not map over their whole length. In such cases, we kept the contig only the plasmid with the highest identity to the reference and longest mapping. In some rare cases, we used the same contig twice in the same plasmid as the PacBio reference showed that a sequence was repeated on the plasmid and thus it was not surprising that the Illumina reads from the two repeated regions would be assembled to the same contig; or we used the same contig in two different plasmids if the contig mapped with the same identity in both plasmids, again because the two plasmids had very similar sequences. Final chromosome and plasmid files were created based on the SNPs and indels identified with the program show-snps from package MUMmer [32]⁠ using following rules: for SNPs keep the Illumina allele if all contigs mapping at this position agree, else keep the reference allele if at least one contig also has it, else replace the base by “N”; keep insertions and deletions if and only if all contigs mapping at this position agree, else keep the reference version.

Final files, either made of an unmapped contig or of several contigs mapped to a reference were kept only if the final sequence length was at least 5000 bp and if unambiguous identification of the plasmid was possible thanks to mapping or the presence of a plasmid partition gene (see below). Shorter sequences were not considered as a plasmid and discarded from the final genomes.

The quality of the final reconstructed genomes was further studied by re-mapping the raw Illumina reads to the final genomes. This was done using BWA-MEM algorithm v. 0.7.17-r1188 [88]⁠ and read duplicates that can arise during library preparation by PCR were removed using Picard v. 2.21.6 (http://broadinstitute.github.io/picard). Read manipulation and extraction of coverage data was done with SAMtools v.1.9 [89]⁠. For isolate NT24, the same procedure was repeated using PacBio plasmids to test for the coverage of the fused plasmids cp32–7 + 7 + 11 and cp32–12 + 5 + 6 (Suppl. Fig. 1). The quality of the assembled genomes was tested by comparing the relative standard deviation of the coverage of the raw reads between chromosomes and plasmids, between populations and between types of procedure to obtain the final sequence (full contig, or several contigs mapped to a reference) using Wilcoxon Rank Sum tests (Suppl. Fig. 3). The relative coverage of plasmids were also compared to the main chromosome over all European samples with Wilcoxon Rank Sum tests with *P*-values corrected for multiple testing with Bonferroni-Holm correction. The coverage of each plasmid relative to the chromosome for all European isolates was represented in Supplementary Figure 4.

### Plasmid identification and plasmid partition genes

Final genome elements were named after the PFam32 protein family sequences that they contained. We used BLAST v. 2.8.1 [33, 34]⁠ (algorithm blastn) to identify the presence of plasmid partition genes of the PFam32, 49, 50 and 57–62 families. In a first BLAST round we used as queries the PFam32 genes sequences of *B. burgdorferi* s.s. strains B31, BOL26, JD1 and 118a and *B. afzelii* strain PKo to cover the whole plasmid diversity and the PFam49, 50 and 57–62 of *B. burgdorferi* s.s. strain MM1. We performed the search both on the final assembled genome and on the SPAdes Illumina contigs of each isolate as some plasmids could not be assembled. We then reiterated the BLAST search using as queries all the hits found in the first search. We then removed from the final hit lists presented in Supplementary Table 1 all hits that were shorter than half the length of the references (reference lengths were around 750 bp for PFam32, 550 bp for PFam49 and PFam50 and 900 to 1100 bp for PFam57–62) and that had no open reading frame over at least half of the length of the reference.

### Quantitative PCR for plasmid copy number estimation

We used a qPCR protocol to estimate the copy number of plasmids cp26, lp17 and lp36 relative to the main chromosome following Millan et al. [38]⁠. This was performed on two isolates of strain PBi (named 2418 and 24510) each grown as three biological replicates in MKP medium with standard conditions [86]⁠. DNA was extracted using a Maxwell automatic purification instrument once cell density reached approximately 10^7^ cells/mL. Digestion with the PstI enzyme (NEB R0140S) was done to ensure equal accessibility of linear and circular plasmids during the PCR reaction. Five hundred nanograms of DNA from each extraction were digested for 1 h and 10 min at 37 °C with 0.5 μL of PstI in a final reaction volume of 25 μL, after which the enzyme was inactivated for 20 min at 80 °C. Quantitative PCR primers were designed to be as similar as possible in their specifications in order for them to be used in a single qPCR run. Primer-BLAST [90]⁠ was used to produce primer candidates that did not bind multiple times within the *B. bavariensis* PBi genome (Suppl. Table 2). PCR samples were prepared using 1 μM primer concentrations and 10 ng of DNA using the S7 Fusion Polymerase system according to standard protocol for a final reaction volume of 20 μL (IsoGene Scientific). A two-step PCR program was chosen due to the small sizes of the amplified fragments with a thermocycle of 30 s initialization at 98 °C, followed by 30 cycles of 98 °C denaturation for 5 s and 63 °C annealing for 20 s finishing with an elongation step at 72 °C for 7 min. PCR products were visualized using a 1% agarose gel. All PCR produced the expected product size.

All qPCR runs were run using the SsoAdvanced™ Universal SYBR® Green Supermix (Bio Rad) according to standard protocol on a Bio Rad C1000 Touch™ Thermocycler with the same thermoprofile as the two step PCR described above. For each run, two technical replicates from each biological replicate (*n* = 3) were used for a total of 6 qPCR replicates per isolate. A standard curve was calculated per run for both the plasmid and chromosome primers using standards of known DNA concentration (20, 3.3, 2.5, 2.0, 0.3, and 0.04 ng/μL) made from a DNA pool of all samples. Each standard was run in triplicate for each primer set. A negative control was included for each technical replicate of either unknowns or standards (*n* = 10 per plate). Each run included unknowns and standards for one plasmid (cp26, lp36, lp17) and the main chromosome. Cycle threshold (CT) values were recorded for all samples. Primer efficiencies were then calculated according to standard protocol (Bio Rad) from these standard curves. Plasmid copy numbers were calculated for each technical replicate according to the equation described in [91]⁠.

### Plasmid fusions

We studied the architecture of plasmids lp17, lp28–4, lp28–7 and cp32–1 in detail as different fusions and translocation involving these plasmids were observed. Following Casjens et al. [14]⁠ we defined as a new plasmid subtype, a plasmid sequence that had with respect to the other plasmid subtypes either presence of 400 bp or longer indels or obvious evidence of past interplasmid DNA exchanges (translocations). Casjens et al.’s criteria also involved synteny, but our current annotation contains mostly hypothetical proteins and did not allow us to test for synteny. We used BLAST v. 2.8.1 [33, 34]⁠ (algorithm blastn) between each of these four plasmids to identify plasmid types.

### Genome annotation

Genome annotation was performed with RAST Annotation Server v. 2.0 [39, 40]⁠ with default parameters. As an annotation is available online for the main chromosome of reference strain PBi (GenBank accession number CP000013), we compared this annotation with the one obtained for our genome reconstruction of strain PBi based on combining PacBio and Illumina data with The SEED Viewer v. 2.0 [40]⁠ and produced a Blast Dot Plot shown in Supplementary Figure 6.

For each one of the 33 isolates, we compared one by one all genes for which the product is at least 50 amino-acids long, with all genes of the 32 others using blastp algorithm from BLAST v. 2.8.1 [33, 34]⁠. We kept all hits that were at least half as long as the query and shared at least 90% sequence identity with the query and recorded on which genomic segment they were located for each isolate.

### Phylogeny reconstruction

Phylogeny reconstruction was performed on the main chromosome as it is known to be very stable in the *B. burgdorferi* s.l. species complex [17]⁠. In addition to the 33 isolates published in this study we also used four *B. bavariensis* strains published in GenBank (under accession numbers CP000013 for strain PBi from Germany, CP003151 for strain BgVir from Russia and CP003866 and CP007564 for strains NMJW1 and SZ from China) and the *B. garinii* strain 20047 as an outgroup to root the tree (GenBank accession number CP028861). Alignment was performed with MAFFT v7.407 [46, 47]⁠ and phylogeny reconstruction was performed with BEAST v1.8.0 [41]⁠ with the following parameters: coalescent model with exponential growth based on doubling time, lognormal-relaxed clock [42]⁠, GTR substitution model [43]⁠. The chain was run for 100 Million steps in three independent runs and convergence was checked with Tracer v. 1.4 [92]⁠. One of the runs did not converge and for the other two runs a burn-in of 30 and 40% respectively was found appropriate. We then used TreeAnnotator v. 1.10.4 [41]⁠ to identify the best tree after burn-in. The phylogeny presented in Fig. 4 was plotted with FigTree v. 1.4.4 (http://tree.bio.ed.ac.uk/software/figtree/). On the phylogeny we added for each branch the gain or loss of plasmids based on the genome reconstructions presented in Table 1 and Supplementary Table 1 (we considered a plasmid as present when either its sequence or one of its specific plasmid partition gene was present) and using maximum parsimony principle. When two solutions leaded to the same minimum number of events, we chose the solution with the lowest number of gains.

Phylogenies were also reconstructed on plasmid cp26 cutting out the *ospC* locus (200 bp upstream and downstream the gene) and on gene *ospC* with BEAST v. 1.8.0 [41]⁠ using the same priors and the same procedures as above except that the coalescent model did not include exponential growth. We included GenBank strains BgVir (*B. bavariensis* CP003201.1), Far04 and PBr (*B. garinii* CP001319.1 and CP001305.1), PKo, K78 and ACA-1 (*B. afzelii* CP002934.1, CP009060.1, CP001250.1) and A14S (*B. spielmanii* CP001467.1). The sequences were aligned with MAFFT v7.407 [46, 47]⁠ and the chains were run for 500 million states for cp26 and 20 million states for *ospC* each in triplicate. Best trees were reconstructed after removing a burn-in of 10% of the chain and all three runs showed very similar results for each tree. Both trees were plotted using FigTree v. 1.4.4 (http://tree.bio.ed.ac.uk/software/figtree/) and manually rotated to produce Fig. 5 comparing *ospC* and plasmid cp26.

### Statistical analyses and genetic diversity

All statistical analyses were performed with R v. 3.5.2 [93]⁠ and genetic distance and genetic diversity were estimated using packages pegas v. 0.12 [94]⁠ and hierfstat v. 0.04–22 [95]⁠ on orthologous plasmid sequences aligned with MAFFT v7.407 [46, 47]⁠ and along the alignments of the main chromosome as well as plasmids cp26 and lp54 (only segments that could be aligned over their whole length) using windows of 1000 bp sliding every 100 bp.

## Supplementary information

**Additional file 1: Supplementary Figure 1.** Coverage of raw reads mapping on PacBio fused plasmids cp32–7 + 7 + 11 (a) and cp32–12 + 5 + 6 (b) of isolate NT24. Illumina raw reads were mapped with BWA-MEM algorithm v. 0.7.17-r1188 [88]⁠ on PacBio fused plasmids cp32–7 + 7 + 11 (a) and cp32–12 + 5 + 6 (b) of isolate NT24. Regions of low to null coverage (marked in red) show that the fusion is not supported by the short-read data.

**Additional file 2: Supplementary Figure 2.** Comparison of three assemblers for Illumina assembly of 25 *B. bavariensis* isolates. These violin plots compare N50 (a) and total length of contigs (b) obtained with QUAST v. 4.6 [37]⁠ on assemblies performed with SPAdes v. 3.10.1 [31]⁠, SOAPdenovo v. 1.0 [35]⁠ and VelvetOptimizer v. 1.0 [36]⁠.

**Additional file 3: Supplementary Figure 3.** Replicon assembly quality as a function of population, mapping method (a) and type of replicon (b). Illumina raw reads were mapped with BWA-MEM algorithm v. 0.7.17-r1188 [88]⁠ to the final reconstructed genomes and the relative standard deviation of the coverage of the raw reads was used as a measure of assembly quality. We compare here replicons from European (left bars) and Asian (right bars) genomes depending on (a) whether the replicon was made as one contig (pink) or as several contigs mapped to a reference (purple) and on (b) whether it was a chromosome (orange) or a plasmid (blue). Error bars show standard error of the mean. ***: Wilcoxon Rank Sum Test for Europe against Asia, *P*-value < 0.001. Other tests comparing mapping methods (a) and type of replicons (b) were not significant.

**Additional file 4: Supplementary Figure 4.** Coverage ratio of European replicons as a proxy for copy number. Illumina raw reads were mapped with BWA-MEM algorithm v. 0.7.17-r1188 [88]⁠ to the final reconstructed genomes and the ratio of the coverage of each replicon with respect to the chromosome was computed in each European isolate. Error bars show standard error of the mean. Dark blue numbers indicate the number of plasmids of this type in the European sample. Wilcoxon Rank Sum Tests comparing coverage of each plasmid with that of the chromosomes: *P*-Value after Bonferroni-Holm correction *: < 0.05, ***: < 0.001, else: not significant.

**Additional file 5: Supplementary Figure 5.** Schematic representation of plasmid subtypes and fusion/relocation events on lp17, lp28–4, lp28–7 and cp32–1. The different plasmid subtypes (numbered arbitrarily) are represented as black bars. We defined as a new plasmid subtype, a plasmid sequence that had, with respect to the other plasmid subtypes, either presence of 400 bp or longer indels or obvious evidence of past interplasmid DNA exchanges (translocations). We used BLAST v. 2.8.1 [33, 34]⁠ to identify plasmid types and colour-shaded areas represent BLAST hits on the same strand (blue) and inversions (pink). Different shades of color are just used for clarity and have no meaning. Dashed lines represent plasmid fusions. Scale bars above the plots are plasmid lengths in kb. *: specific cases: Arh913 cp32–1 could no be assembled. Konnai17 had two lp28–7 plasmids, the second one has the same subtype as plasmid lp28–7 in FujiP2.

**Additional file 6: Supplementary Figure 6.** Dotplot comparing annotation of strain PBi between our isolate and a previously published one. Comparison of gene content realized in RAST Annotation Server v. 2.0 [39, 40]⁠ on the main chromosome. PBi accession number in RAST: 290434.1.

**Additional file 7: Supplementary Figure 7.** Genetic diversity along the main chromosome of *B. bavariensis.* Genetic diversity was estimated using R package pegas v. 0.12 [94]⁠ on orthologous sequences aligned with MAFFT v7.407 [46, 47]⁠ on 1000 bp windows sliding every 100 bp in Asian isolates only (a), European isolates only (b) and all isolates (c). Genes located on diversity peaks (d) come from RAST Annotation Server v. 2.0 [39, 40]⁠.

**Additional file 8: Supplementary Figure 8.** Genetic diversity along plasmid cp26 of *B. bavariensis.* Genetic diversity was estimated using R package pegas v. 0.12 [94]⁠ on orthologous sequences aligned with MAFFT v7.407 [46, 47]⁠ on 1000 bp windows sliding every 100 bp in Asian isolates only (a), European isolates only (b) and all isolates (c). Genes located on diversity peaks (d) come from RAST Annotation Server v. 2.0 [39, 40]⁠.

**Additional file 9: Supplementary Figure 9.** Genetic diversity along plasmid lp54 of *B. bavariensis.* Genetic diversity was estimated using R package pegas v. 0.12 [94]⁠ on orthologous sequences aligned with MAFFT v7.407 [46, 47]⁠ on 1000 bp windows sliding every 100 bp in Asian isolates only (a), European isolates only (b) and all isolates (c). Genes located on diversity peaks (d) come from RAST Annotation Server v. 2.0 [39, 40]⁠.

**Additional file 10: Supplementary Table 1.** Plasmid partition genes identified in 33 B. bavariensis strains. **Supplementary Table 2.** Primers used for qPCR.

## Data Availability

The datasets generated and/or analyzed during the current study are available in the NCBI repository BioProjects PRJNA449844 and PRJNA327303.

## Abbreviations

LB: Lyme Borreliosis
NGS: Next-generation sequencing
PacBio: Pacific Bioscience SMRT sequencing
SNP: Single nucleotide polymorphism
GTR: General Time Reversible (substitution model)
