# Supplementary figures and images for: High conservation combined with high plasticity: genomics and evolution of Borrelia bavariensis
Source: BMC Genomics. 2020 Oct 8;21:702. doi: 10.1186/s12864-020-07054-3 (PMC7542741; doi:10.1186/s12864-020-07054-3)

**a**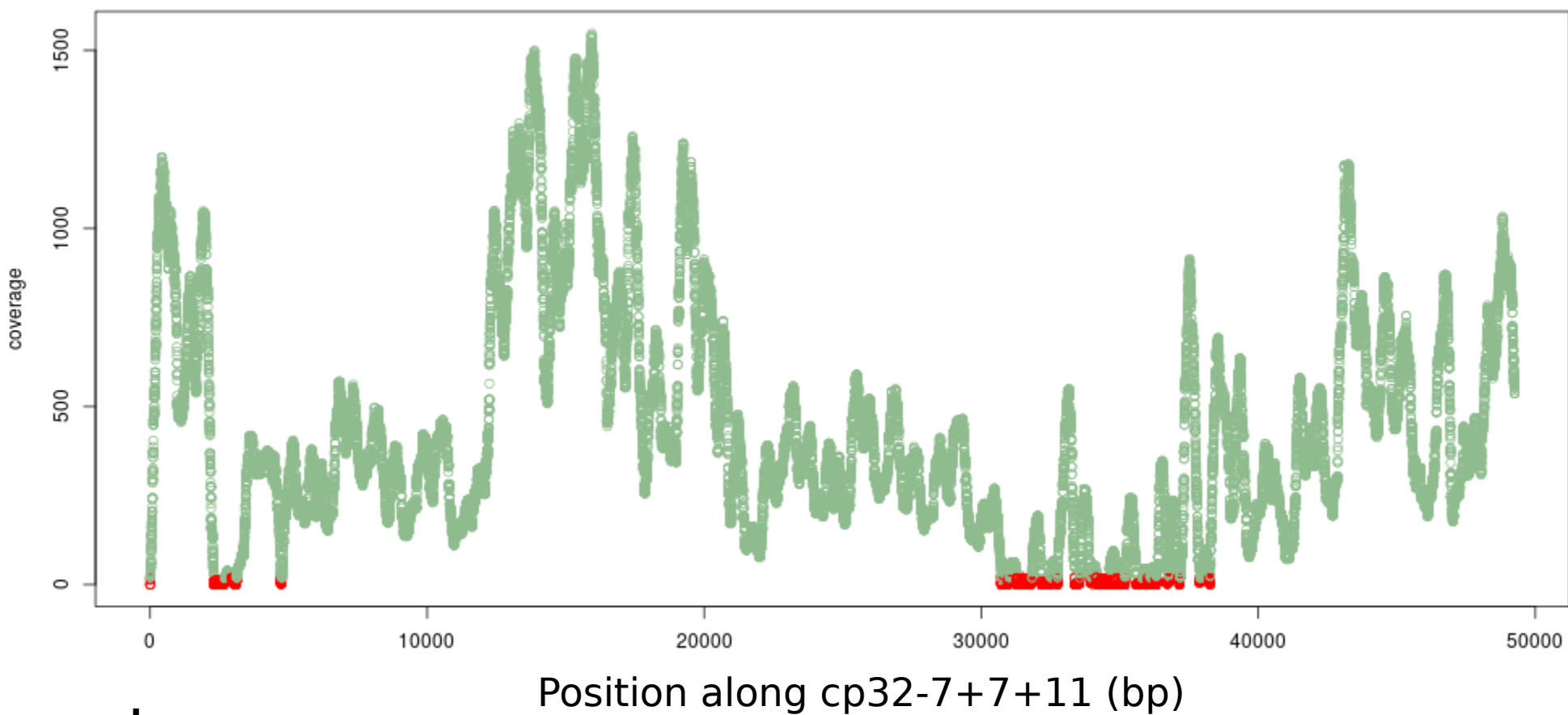**b**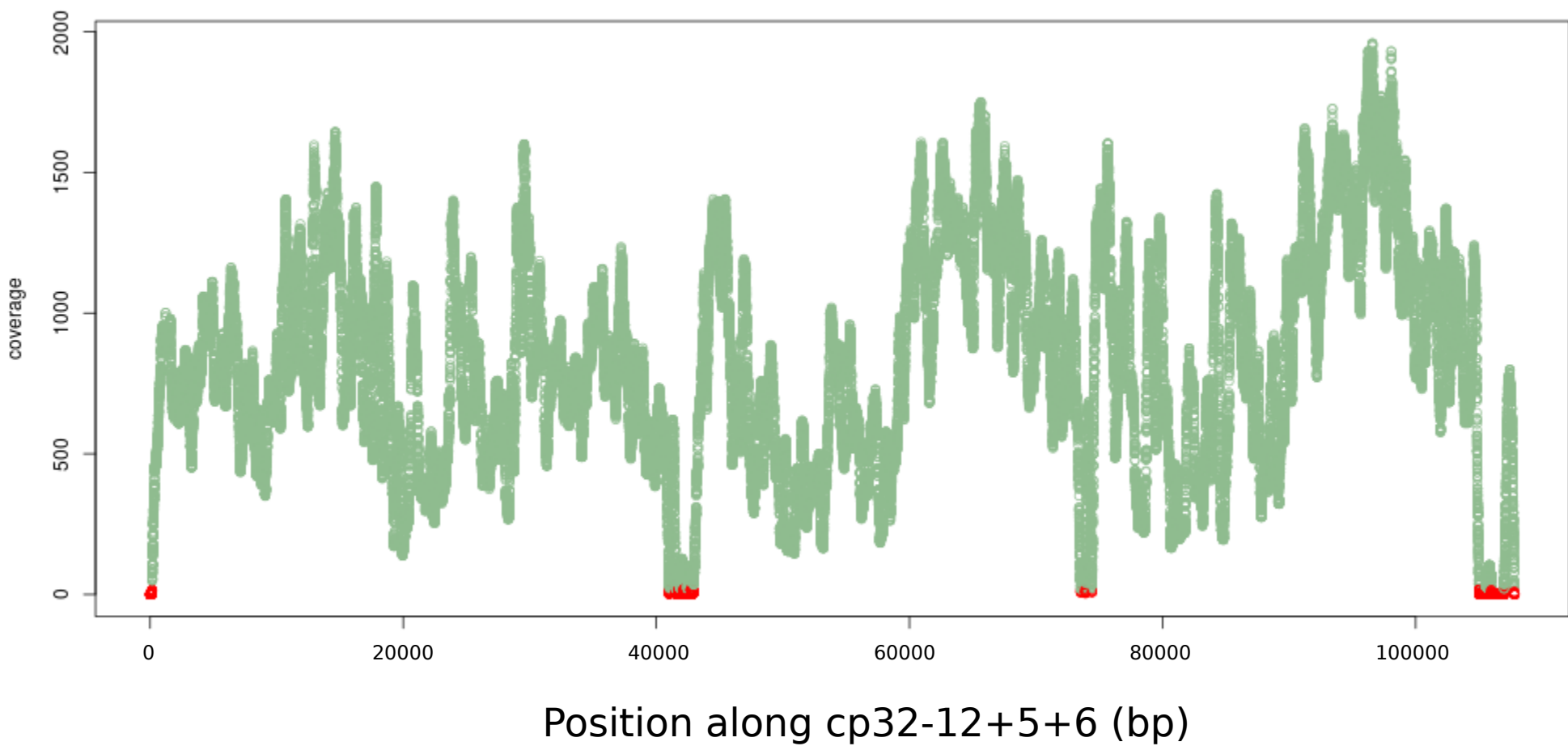

Supplement: Supplementary file 1 — Additional file 1: Supplementary Figure 1. Coverage of raw reads mapping on PacBio fused plasmids cp32–7 + 7 + 11 (a) and cp32–12 + 5 + 6 (b) of isolate NT24. Illumina raw reads were mapped with BWA-MEM algorithm v. 0.7.17-r1188 [88]⁠ on PacBio fused plasmids cp32–7 + 7 + 11 (a) and cp32–12 + 5 + 6 (b) of isolate NT24. Regions of low to null coverage (marked in red) show that the fusion is not supported by the short-read data. [file 12864_2020_7054_MOESM1_ESM.pdf]

**a**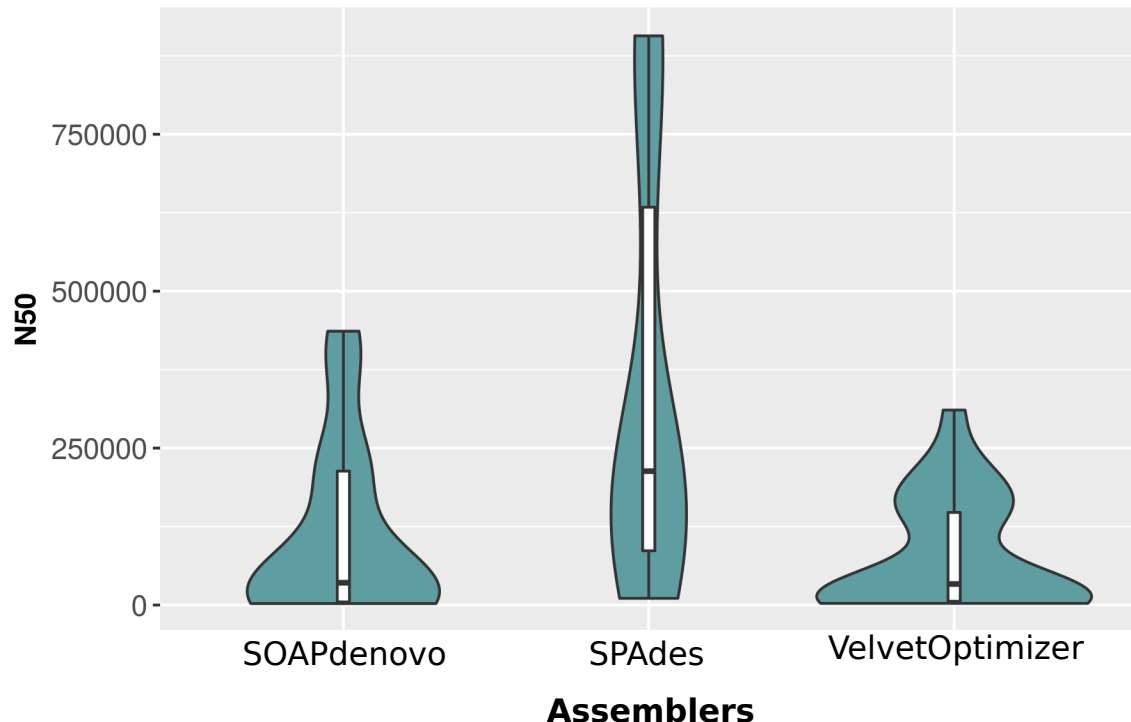**b**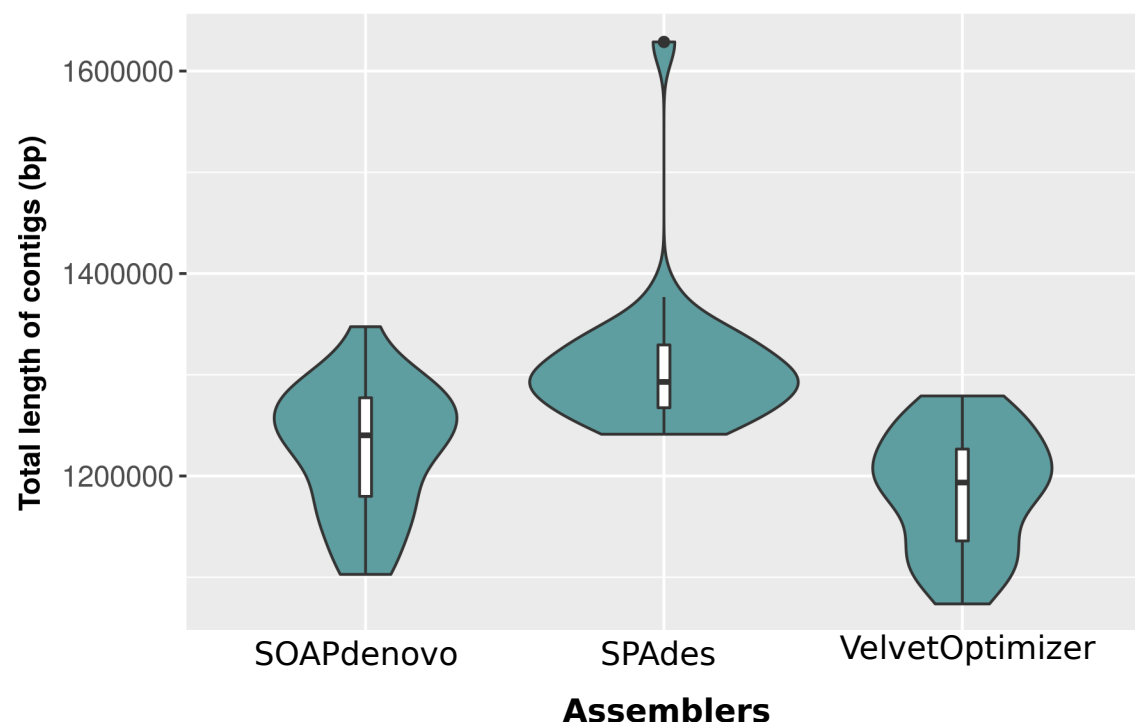

Supplement: Supplementary file 2 — Additional file 2: Supplementary Figure 2. Comparison of three assemblers for Illumina assembly of 25 B. bavariensis isolates. These violin plots compare N50 (a) and total length of contigs (b) obtained with QUAST v. 4.6 [37]⁠ on assemblies performed with SPAdes v. 3.10.1 [31]⁠, SOAPdenovo v. 1.0 [35]⁠ and VelvetOptimizer v. 1.0 [36]⁠. [file 12864_2020_7054_MOESM2_ESM.pdf]

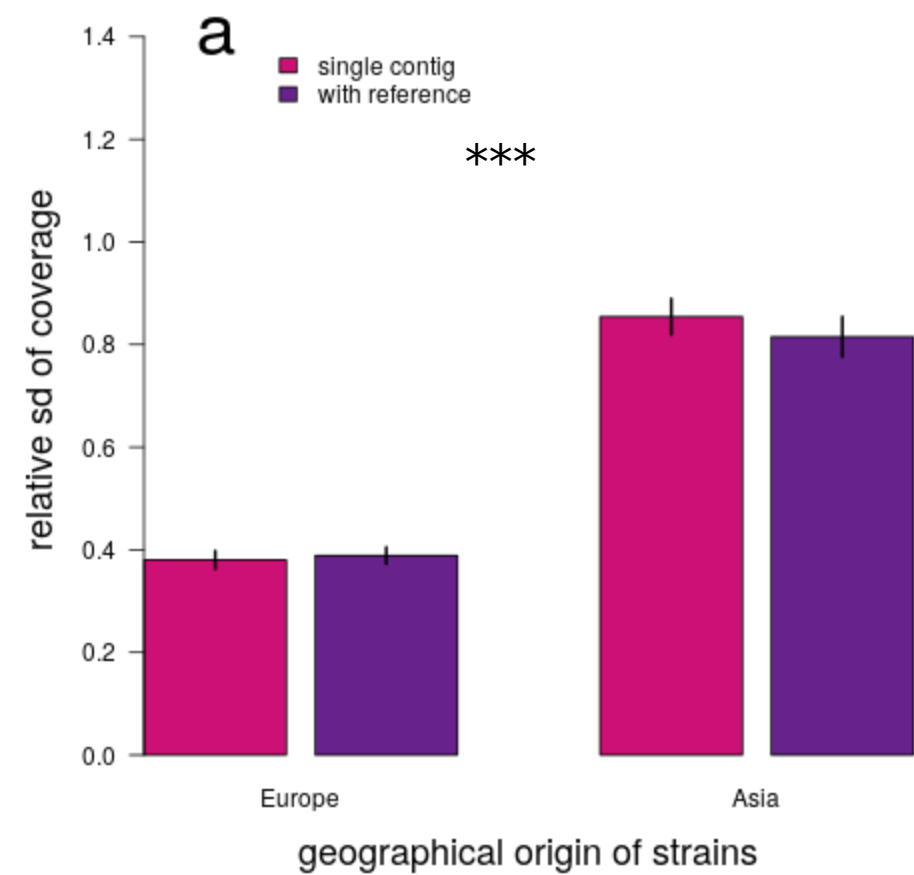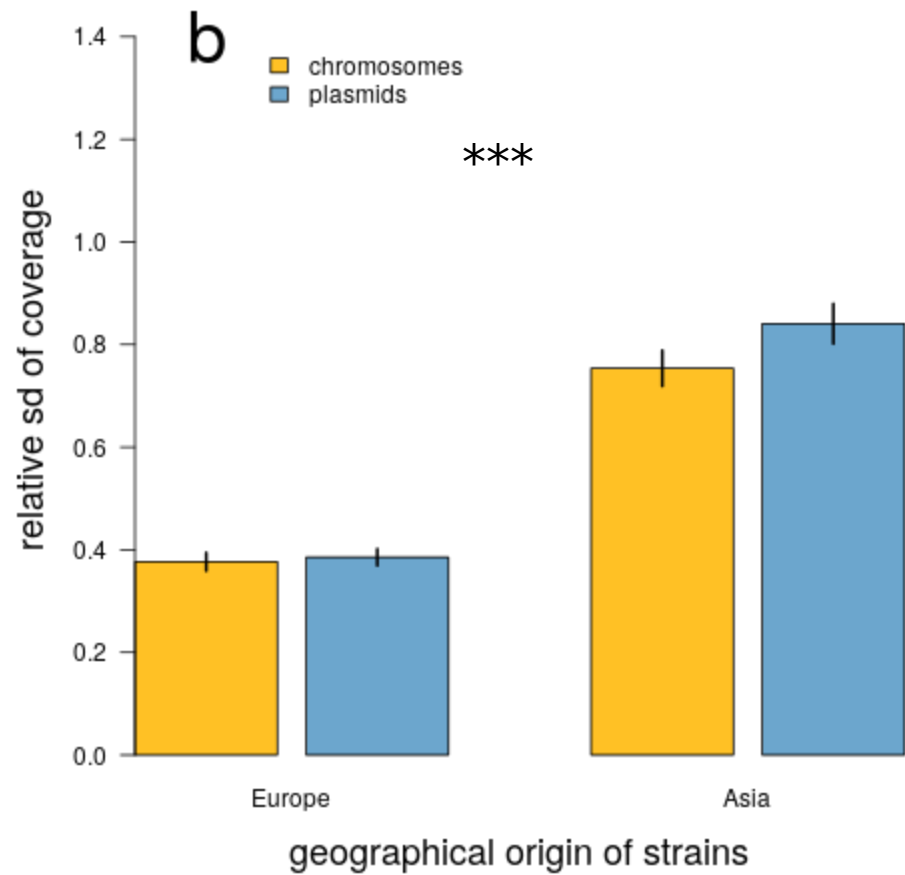

Supplement: Supplementary file 3 — Additional file 3: Supplementary Figure 3. Replicon assembly quality as a function of population, mapping method (a) and type of replicon (b). Illumina raw reads were mapped with BWA-MEM algorithm v. 0.7.17-r1188 [88]⁠ to the final reconstructed genomes and the relative standard deviation of the coverage of the raw reads was used as a measure of assembly quality. We compare here replicons from European (left bars) and Asian (right bars) genomes depending on (a) whether the replicon was made as one contig (pink) or as several contigs mapped to a reference (purple) and on (b) whether it was a chromosome (orange) or a plasmid (blue). Error bars show standard error of the mean. ***: Wilcoxon Rank Sum Test for Europe against Asia, P-value < 0.001. Other tests comparing mapping methods (a) and type of replicons (b) were not significant. [file 12864_2020_7054_MOESM3_ESM.pdf]

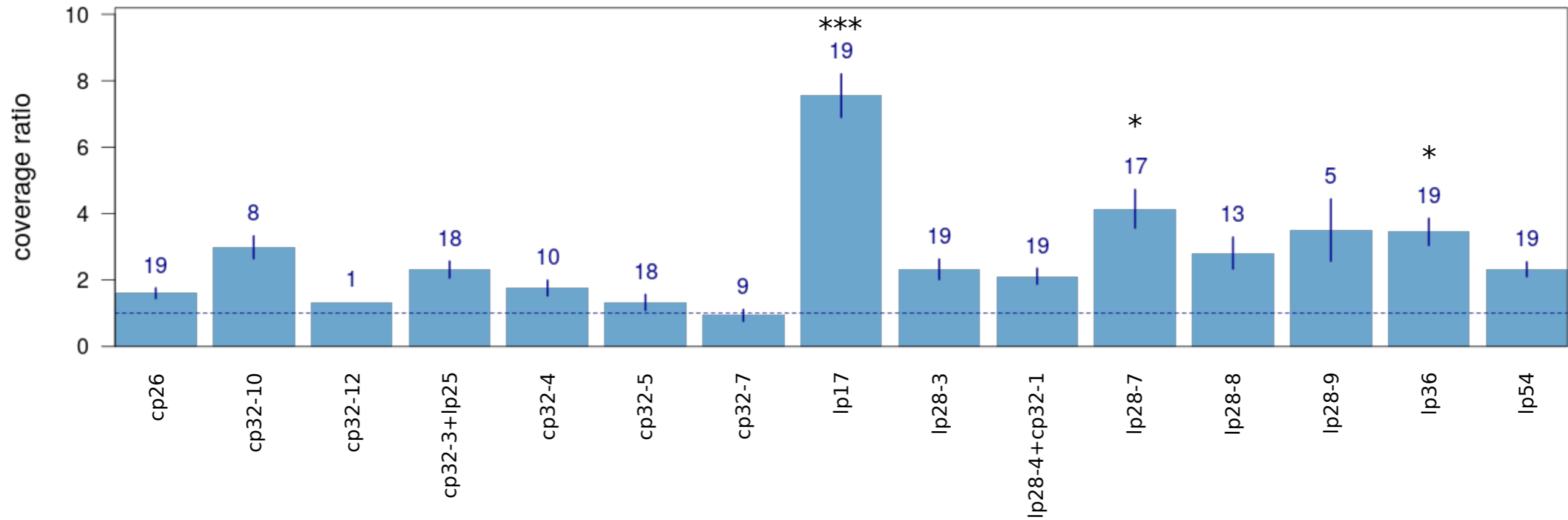

Supplement: Supplementary file 4 — Additional file 4: Supplementary Figure 4. Coverage ratio of European replicons as a proxy for copy number. Illumina raw reads were mapped with BWA-MEM algorithm v. 0.7.17-r1188 [88]⁠ to the final reconstructed genomes and the ratio of the coverage of each replicon with respect to the chromosome was computed in each European isolate. Error bars show standard error of the mean. Dark blue numbers indicate the number of plasmids of this type in the European sample. Wilcoxon Rank Sum Tests comparing coverage of each plasmid with that of the chromosomes: P-Value after Bonferroni-Holm correction *: < 0.05, ***: < 0.001, else: not significant. [file 12864_2020_7054_MOESM4_ESM.pdf]

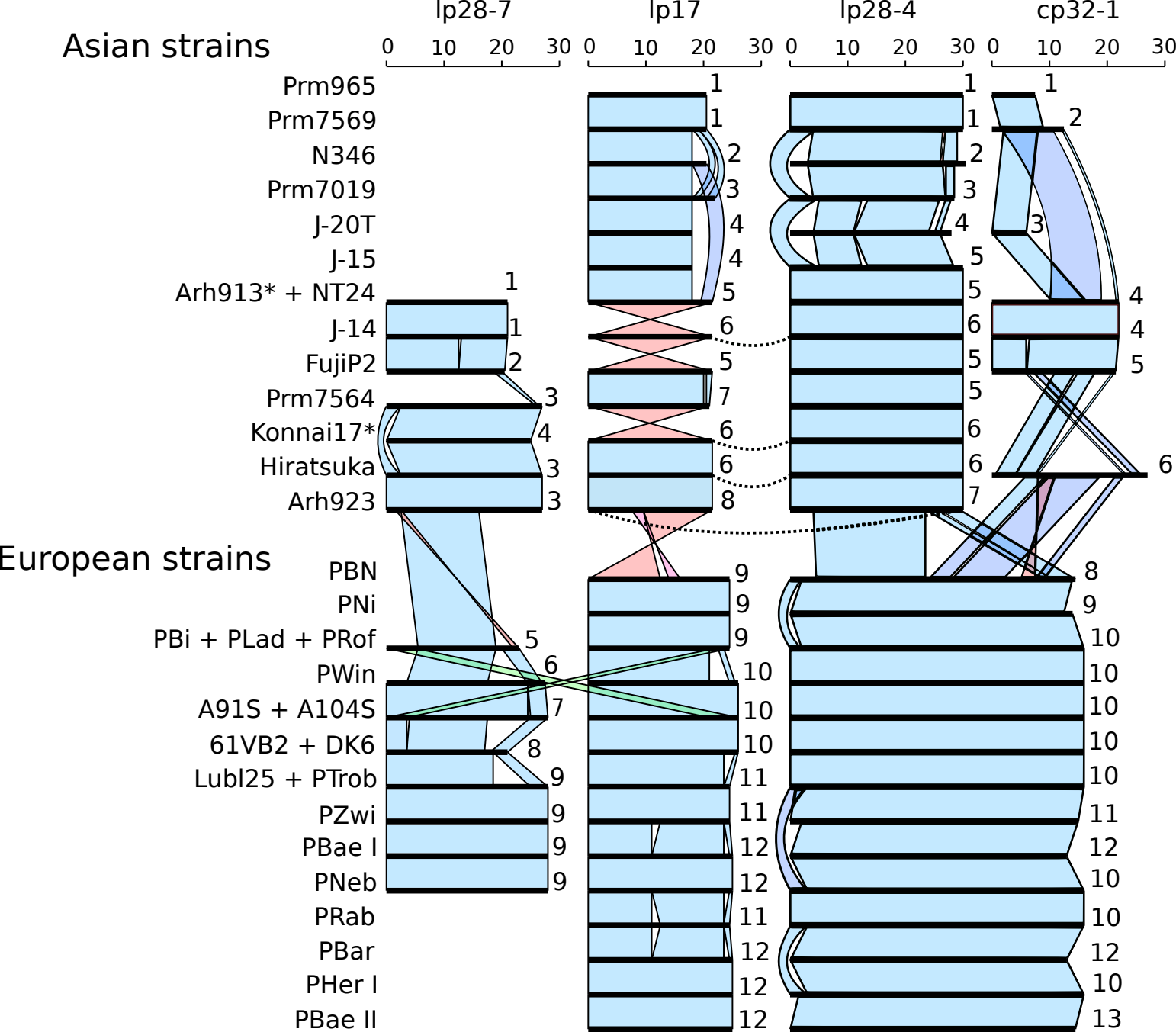

Supplement: Supplementary file 5 — Additional file 5: Supplementary Figure 5. Schematic representation of plasmid subtypes and fusion/relocation events on lp17, lp28–4, lp28–7 and cp32–1. The different plasmid subtypes (numbered arbitrarily) are represented as black bars. We defined as a new plasmid subtype, a plasmid sequence that had, with respect to the other plasmid subtypes, either presence of 400 bp or longer indels or obvious evidence of past interplasmid DNA exchanges (translocations). We used BLAST v. 2.8.1 [33, 34]⁠ to identify plasmid types and colour-shaded areas represent BLAST hits on the same strand (blue) and inversions (pink). Different shades of color are just used for clarity and have no meaning. Dashed lines represent plasmid fusions. Scale bars above the plots are plasmid lengths in kb. *: specific cases: Arh913 cp32–1 could no be assembled. Konnai17 had two lp28–7 plasmids, the second one has the same subtype as plasmid lp28–7 in FujiP2. [file 12864_2020_7054_MOESM5_ESM.pdf]

*Borrelia bavariensis* PBi our data

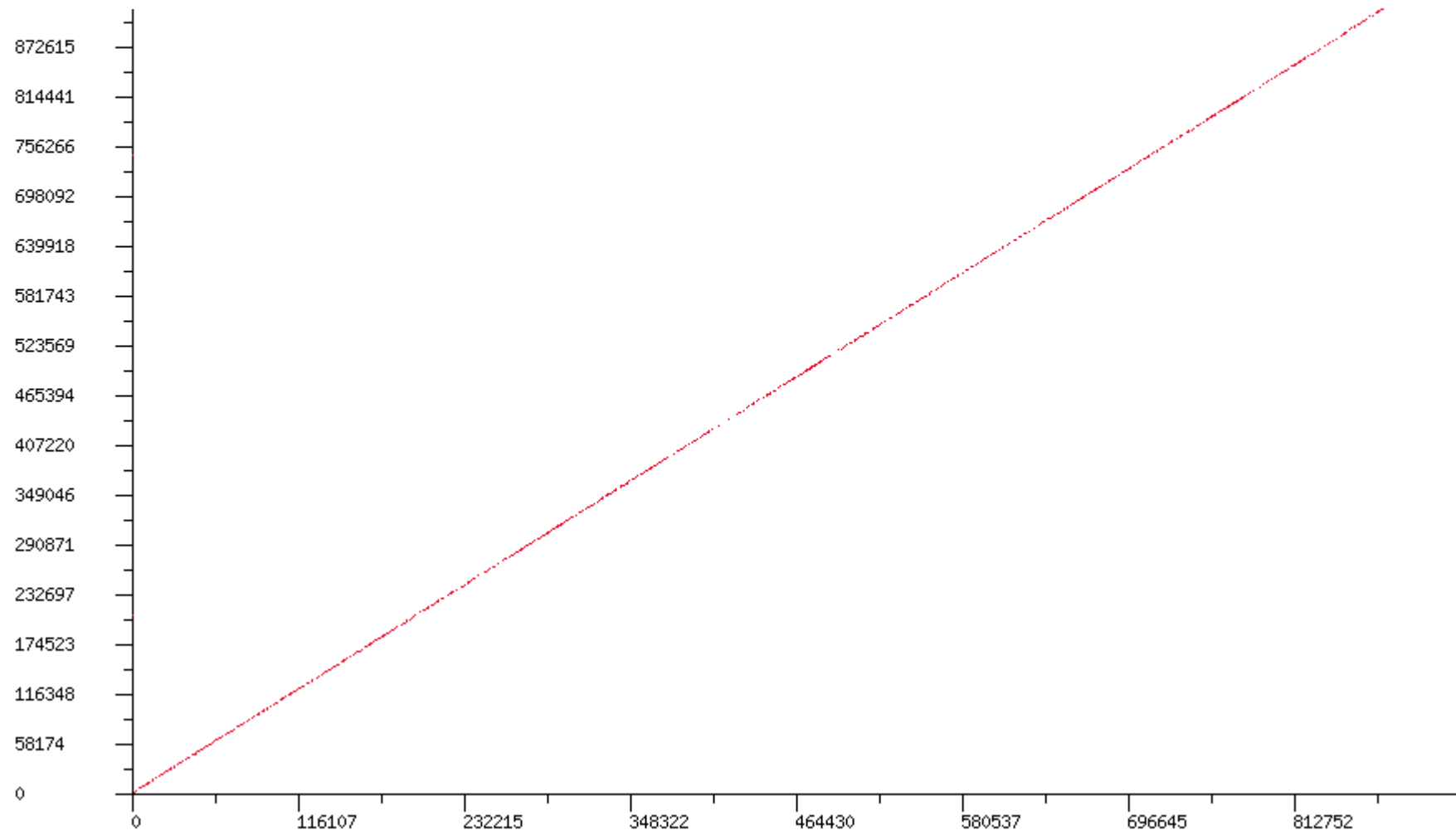

Supplement: Supplementary file 6 — Additional file 6: Supplementary Figure 6. Dotplot comparing annotation of strain PBi between our isolate and a previously published one. Comparison of gene content realized in RAST Annotation Server v. 2.0 [39, 40]⁠ on the main chromosome. PBi accession number in RAST: 290434.1. [file 12864_2020_7054_MOESM6_ESM.pdf]

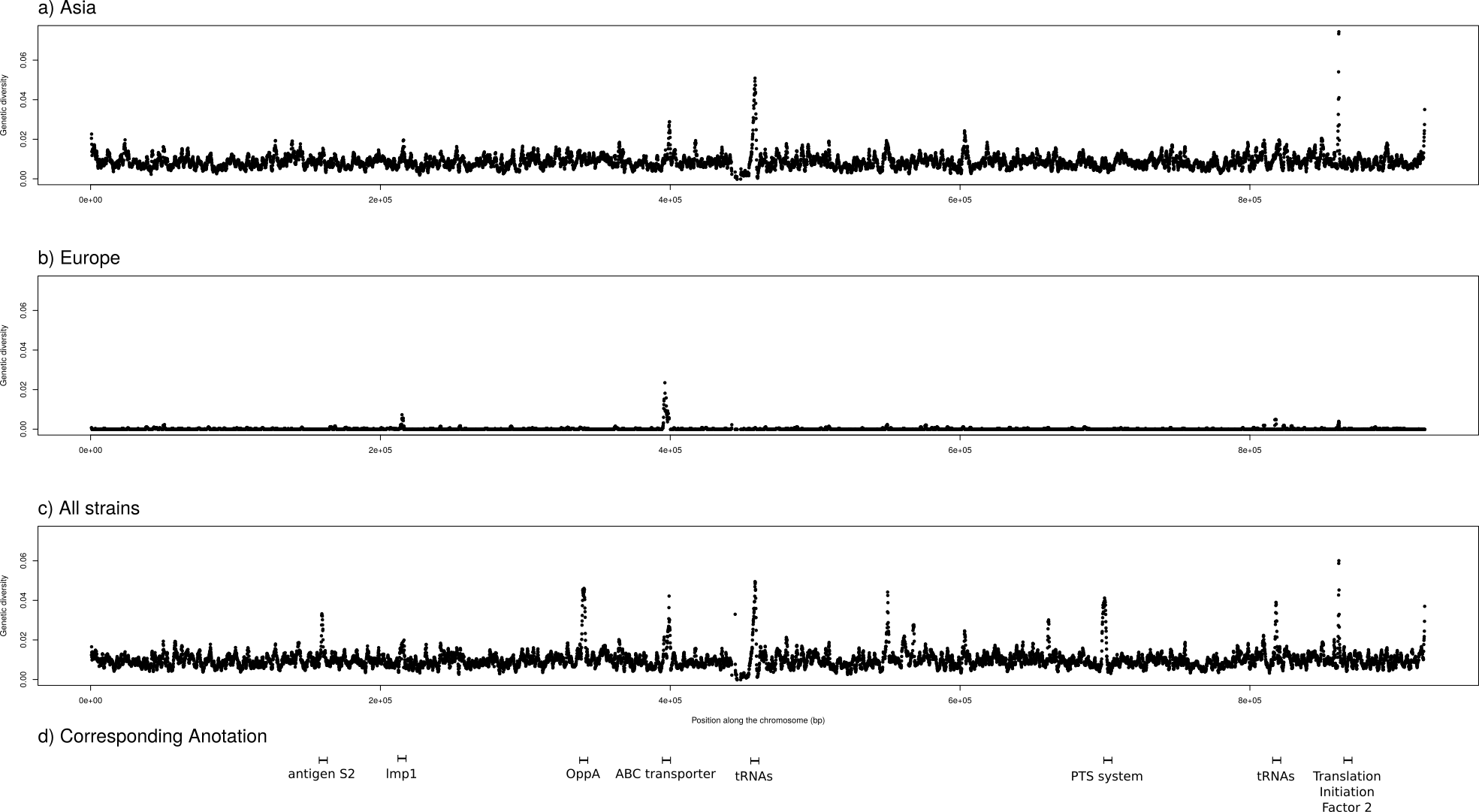

Supplement: Supplementary file 7 — Additional file 7: Supplementary Figure 7. Genetic diversity along the main chromosome of B. bavariensis. Genetic diversity was estimated using R package pegas v. 0.12 [94]⁠ on orthologous sequences aligned with MAFFT v7.407 [46, 47]⁠ on 1000 bp windows sliding every 100 bp in Asian isolates only (a), European isolates only (b) and all isolates (c). Genes located on diversity peaks (d) come from RAST Annotation Server v. 2.0 [39, 40]⁠. [file 12864_2020_7054_MOESM7_ESM.png]

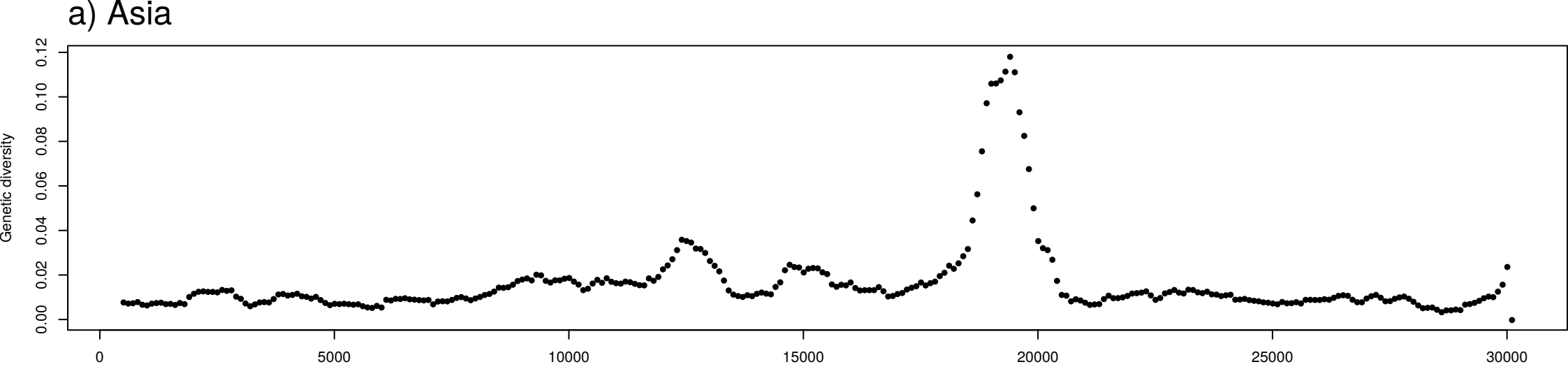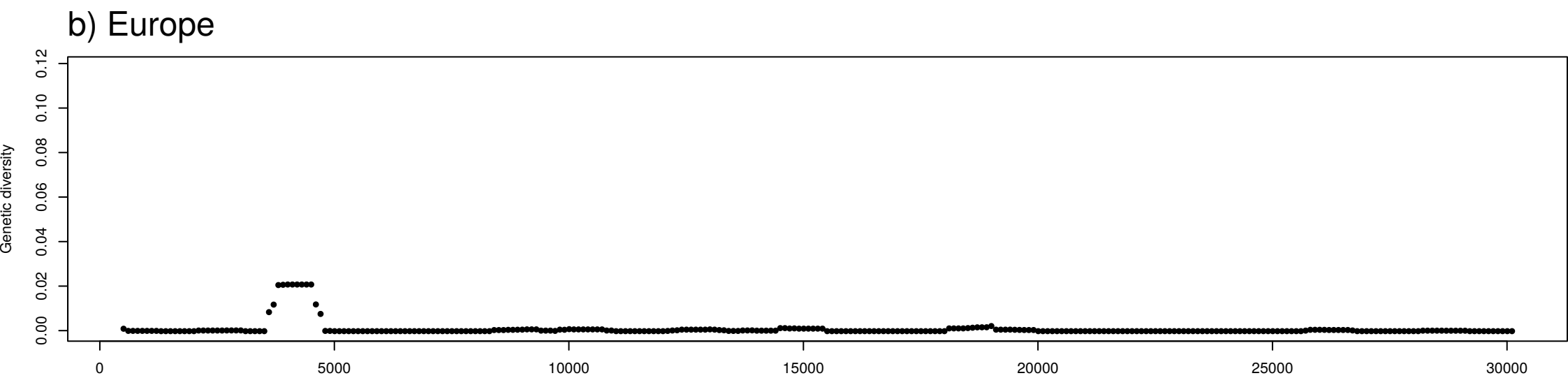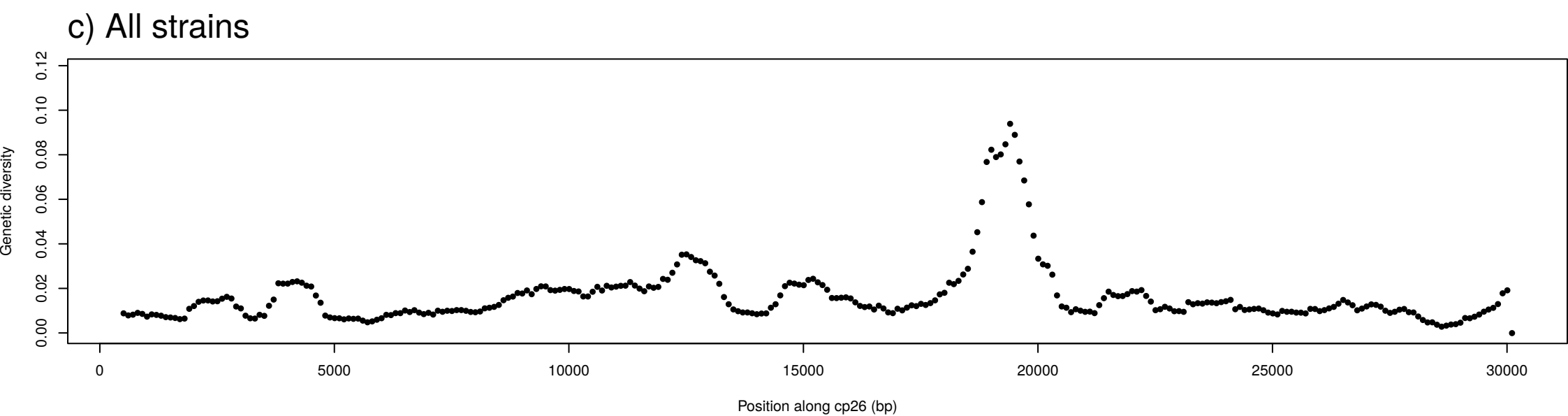

d) Corresponding Anotation

PTS system  
chitibiose-specific  
components

OspC

Supplement: Supplementary file 8 — Additional file 8: Supplementary Figure 8. Genetic diversity along plasmid cp26 of B. bavariensis. Genetic diversity was estimated using R package pegas v. 0.12 [94]⁠ on orthologous sequences aligned with MAFFT v7.407 [46, 47]⁠ on 1000 bp windows sliding every 100 bp in Asian isolates only (a), European isolates only (b) and all isolates (c). Genes located on diversity peaks (d) come from RAST Annotation Server v. 2.0 [39, 40]⁠. [file 12864_2020_7054_MOESM8_ESM.pdf]

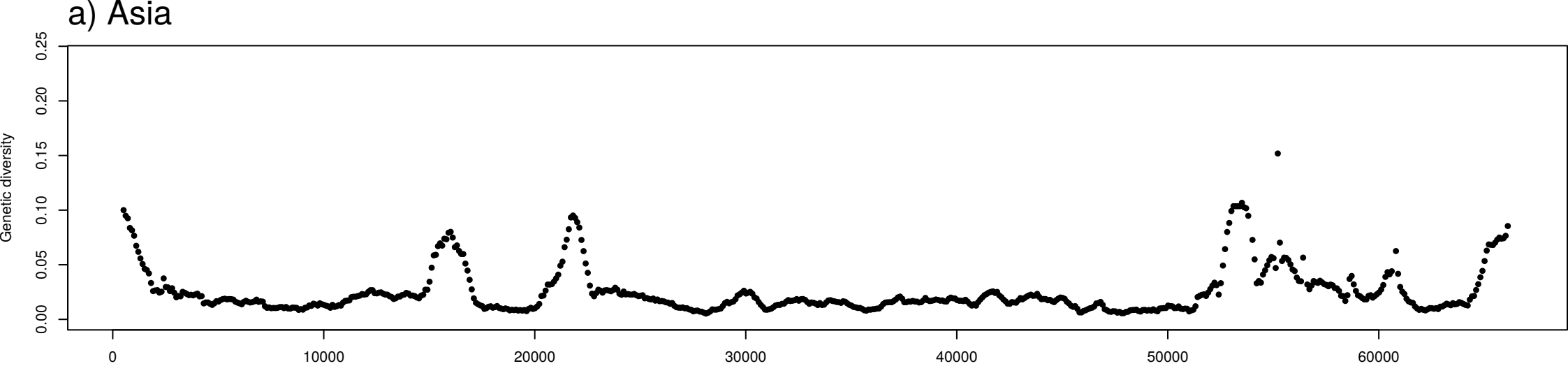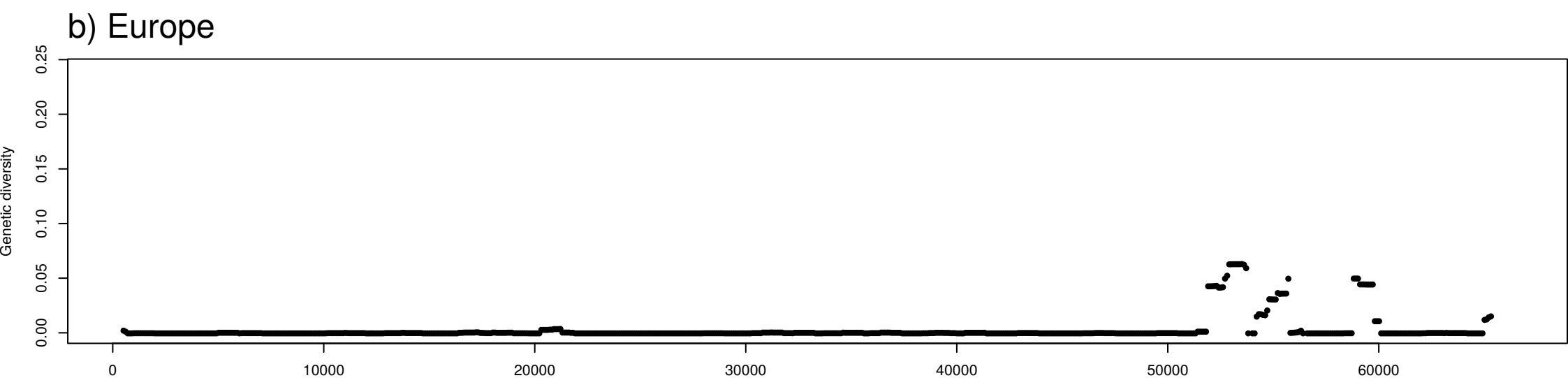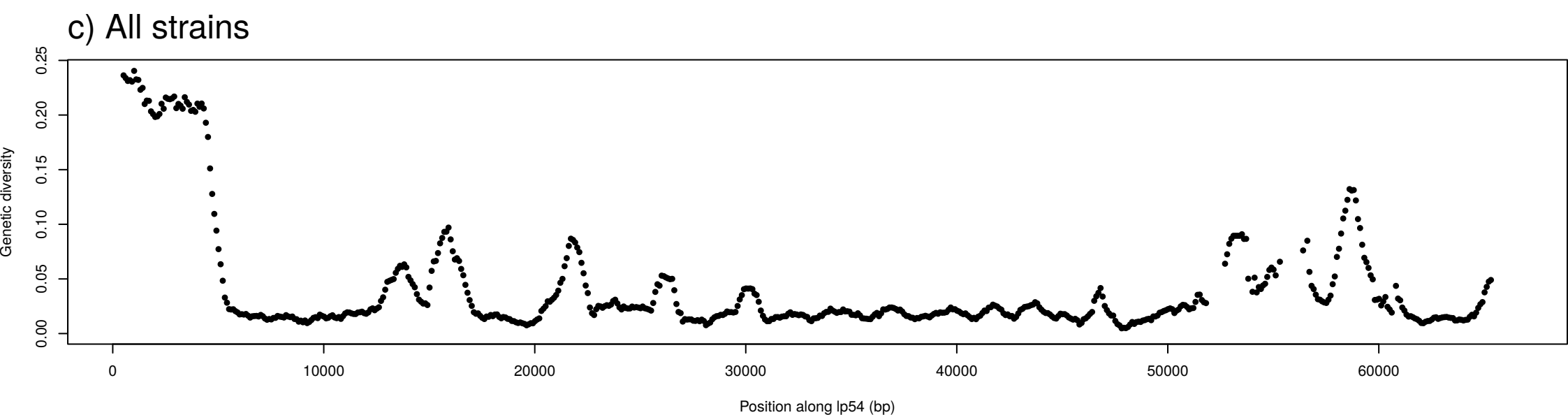

d) Corresponding Anotation

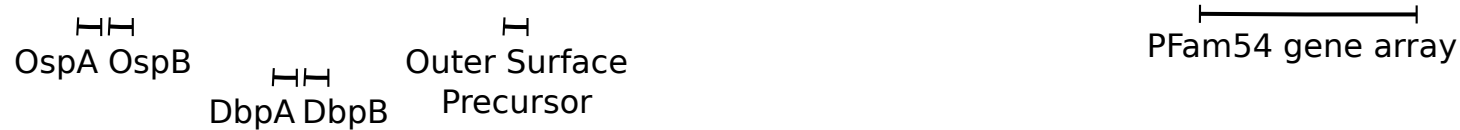

Supplement: Supplementary file 9 — Additional file 9: Supplementary Figure 9. Genetic diversity along plasmid lp54 of B. bavariensis. Genetic diversity was estimated using R package pegas v. 0.12 [94]⁠ on orthologous sequences aligned with MAFFT v7.407 [46, 47]⁠ on 1000 bp windows sliding every 100 bp in Asian isolates only (a), European isolates only (b) and all isolates (c). Genes located on diversity peaks (d) come from RAST Annotation Server v. 2.0 [39, 40]⁠. [file 12864_2020_7054_MOESM9_ESM.pdf]
