# Supplementary material for: High conservation combined with high plasticity: genomics and evolution of Borrelia bavariensis
Source: BMC Genomics. 2020 Oct 8;21:702. doi: 10.1186/s12864-020-07054-3 (PMC7542741; doi:10.1186/s12864-020-07054-3)
Supplement: Supplementary file 10 — Additional file 10: Supplementary Table 1. Plasmid partition genes identified in 33 B. bavariensis strains. Supplementary Table 2. Primers used for qPCR. [file 12864_2020_7054_MOESM10_ESM.pdf]

Strain	cp26	lp54	lp36	lp17	lp17+lp28-4	lp25	lp38	lp56	lp28-2	lp28-3	lp28-4	lp28-4+cp32-1	lp28-6	lp28-7	lp28-8	lp28-9	cp32-1	cp32-3	cp32-3+lp25	cp32-4	cp32-5	cp32-6	cp32-7	cp32-9	cp/lp32-10	cp32-11	cp32-12
Arh913	32/49/57	32/49/50/57	32/49/50/57	32		32/49/50/57	32/49/50			32/49/57	32/49/50/57			57	32/49/50		32/49			32/49/57		32/57	32/49/50/57	32	32/49/50/57	32/49/57	32/49/50
Arh923	32/49/57	32/49/50/57	32/49/50/57		32/49/50/57 *	32/49/50/57	32/49/50			32/49/50/57			57	32		32		32/49/50/57		32/49/50/57	32/49/57			32/49/50/57	32/49/50/57	32/49/57	
FujiP2	32/49/57	32/49/50/57	32/49/50/57	32		32/49/50/57				32/49/57	32/49/50/57		57	57	32/49/50		32/49/50/57			32/49/57	32/49/57	32	32/49/50/57	32/49/50/57	32/49/50/57	32/49/57	32/49/50/57
Hiratsuka	32/49/57	32/49/50/57	32/49/50/57		32/49/50/57 *	32/49/50/57		32		32/49/50/57			57	32		32	32/49/50/57	32/49/50/57					32/49/50/57	32/49/50/57	32/49/50/57	32/49/57	
J-14	32/49/57	32/49/50/57	32/49/50/57		32/49/50/57 *	32/49/50/57				32/49/57			57	57	32/49/50		32/49/50/57			32/57		32/50/57	32/49/50/57		32/49/50/57	32/49/57	32/49/50/57
J-15	32/49/57	32/49/50/57	32/49/50/57	32		32/49/50/57	32/49/50		32/57	32/49/50/57	32/49/50/57									32/49		32	32/49		32/49/50/57		
J-20T	32/49/57	32/49/50/57	32/49/50/57	32		32/49/50/57					32/49/50/57		57			32	32/49/50/57	32/49/57		32/49/50/57	32/49/50/57		32/49/50/57		32/49/57	32/49/57	32/49/50/57
Konnai17	32/49/57	32/49/50/57	32/49/50/57		32/49/50/57 *		32/49						57	32 + 57 *			32/49/50/57			32/49/57		32/50/57	32/49	32/49/50/57	32/49/50/57	32/49/57	32/49/50/57
N346	32/49/57	32/49/50/57	32/49/50/57	32		32/49/50/57				32/49/57					32/49/50			32/49/57			32/49/57	32/50/57	32/49/50/57		32/49/50/57	32/49/57	49/57
NT24	32/49/57	32/49/50/57	32/49/50/57	32		32/49/50/57				32/49/57	32/49/50/57			57	32/49/50		32/49/50/57			32/49/57	32/49/57	32/50/57	32/49/50/57	32/49/50/57	32/49/50/57	32/49/57	32/49/50/57
Prm7019	32/49/57	32/49/50/57	32/49/50/57	32		32/49/50/57	32/49/50			32/49/57	32/49/50/57				32/49/50						32/49/57		32/49/50	32/49/50/57	32/49/50/57	32	32/49/50/57
Prm7564	32/49/57	32/49/50/57	32/49/50/57	32		32/49/50/57	32/49/50			32/49/50/57	32/49/50/57		57	32				32/49/50/57		32/49/50/57	32/49/57			32/49/50/57	32/49/50/57	32/49/57	
Prm7569	32/49/57	32/49/50/57	32/49/50/57	32		32/49/50/57	32/49/50			32/49/57	32/49/50/57			32	32/49/50		32/49/50/57			32/49/50/57	32/49/57	32	32/49/50	32/49/50/57	32/49/50/57		32/49/57
Prm965	32/49/57	32/49/50/57	32/49/50/57	32		32/49/57					32/49/50/57				32/49/50	32	32/49/50/57	32/49/50		32/49/50/57	32/49/57	32	32	32/49	57		32/49/57
61VB2	32/49/57	32/49/50/57	32/49/50/57	32						32/49/57		32/49/50/57		32	32/49/50				32/49/57		32/49/57		32/49/50/57				
A104S	32/49/57	32/49/50/57	32/49/50/57	32						32/49/57		32/49/50/57		32	32/49/50				32/49/57 *	32/49	32/49		32/49/50/57		32/49/50/57		
A91S	32/49/57	32/49/50/57	32/49/50/57	32						32/49/57		32/49/50/57		32	32/49/50				32/49/57	32/49/50/57	32/49/57		32/49/50/57				
DK6	32/49/57	32/49/50/57	32/49/50/57	32						32/49/57		32/49/50/57		32	32 50				32/49/57		32/49/57		32/49/50/57				
Lubl25	32/49/57	32/49/50/57	32/49/50/57	32						32/49/57		32/49/50/57		32	32/49/50				32/49/57	32/49/50/57	32/49/57				32/49/50/57		
PBae I	32/49/57	32/49/50/57	32/49/50/57	32						32/49/50/57		32/49/50/57		32					32/49/57	32/49/50/57	32/49/57						
PBae II	32/49/57	32/49/50/57	32/49/50/57	32						32/49/57		32/49/50/57		32	32/49/50				32/49/50/57 *	32/49/50/57	32/49						
PBar	32/49/57	32/49/50/57	32/49/50/57	32						49/57 *		32/49/50/57		32	32/49/50				32/49/57	32/49/50/57	32/49/57				32/49/50/57		
PBi	32/49/57	32/49/50/57	32/49/50/57	32						32/49/57		32/49/50/57		32	50	32			32/49/57		32/49/57		32/49/50/57				
PBN	32/49/57	32/49/50/57	32/49/50/57	32						32/49/57		32/49/50/57				32			32/49/57		32/49/57		32/49/50/57				32/50/57
PHer I	32/49/57	32/49/50/57	32/49/50/57	32						32/49/57		32/49/50/57		32	32/49/50				32/49/57	32/49/50/57	32/49/57				32/49/50/57		
PLad	32/49/57	32/49/50/57	32/49/50/57	32						32/49/57		32/49/50/57		32	32/49/50	32			32/49/57		32/49/57		32/49/50/57				
PNeb	32/49/57	32/49/50/57	32/49/50/57	32						32/49/57		32/49/50/57		32	32/49/50				32/49/57	32/49/50/57	32/49/57				32/49/50/57		
PNi	32/49/57	32/49/50/57	32/49/50/57	32						32/49/57		32/49/50/57				32			32/49/57		32/49/57		32/49/50/57				
PRab	32/49/57	32/49/50/57	32/49/50/57	32						32/49/57		32/49/50/57		32	32/49/50				32/49/57	32/49/50/57	32/49/57						
PRof	32/49/57	32/49/50/57	32/49/50/57	32						32/49/57		32/49/50/57		32	32/49/50	32			32/49/57		32/49/57		32/49/50/57				
PTrob	32/49/57	32/49/50/57	32/49/50/57	32						32/49/57		32/49/50/57		32	32/49/50				32/49/57	32/49/50/57	32/49/57				32/49/50/57		
PWin	32/49/57	32/49/50/57	32/49/50/57	32						32/49/57		32/49/50/57		32	32/49/50				32/49/50/57 *	32/49/50/57	32/49/57				32/49/50/57		
PZwi	32/49/57	32/49/50/57	32/49/50/57	32						32/49/57		32/49/50/57		32					32/49/57 *	32/49/57	32/49				32/49/50/57		

Supplementary Table 1. Plasmid partition genes identified in 33 *B. bavariensis* strains

Plasmid partition genes were searched for using BLAST v. 2.8.1 on assembled plasmids and on Illumina contigs. The queries were sequences from *B. burgdorferi* sensu stricto strains B31, BOL26, JD1, 118a and MM1 and *B. afzelii* PKo for PFam protein families 32, 49, 50 and 57. All hits on the *B. bavariensis* genomes were then subsequently used as query for a second BLAST run. Final hits that were shorter than half the original gene sequence were removed. Gray cells represent a reconstructed plasmid of at least 5 kb length (shown in Table 1).

* specific cases:

lp17l+p28-4 fusion: lp17 part has only one PFam32 hit whereas the lp28-4 part has all four PFam;

lp28-3 PBar: the PFam32 hit was present but not full as stretched over two contigs;

lp28-7 Konnai17: Konnai17 has two lp28-7 plasmids, one carrying PFam32 and one PFam57;

cp32-3lp25: one European strain had no PFam49 for cp32-3 (A104S) and three others had two lp25 hits for PFam50 (PBae II, PWin, PZwi).

Target	Sense	Sequence (5'-3')	Size (bp)	Tm (°C)	Product size (bp)
chromosome	F	gaaagcaaggcaacaaggg	19	57	133
	R	tgcccctttgagcttacagaag	21	59	
cp26	F	tcacaccagaaagtgcaagc	20	58	130
	R	cctcccattacgctcatttg	20	58	
lp17	F	gaagggctaacggcactcaa	20	60	132
	R	agatgtgaaggaggagcatca	21	59	
lp36	F	cgggtgcattagagcaggat	20	60	127
	R	accaatagcaccccacgttt	20	58	

Supplementary Table 2. Primers used for qPCR
